# Interferon-induced transmembrane protein 3 blocks fusion of sensitive but not resistant viruses by partitioning into virus-carrying endosomes

**DOI:** 10.1371/journal.ppat.1007532

**Published:** 2019-01-14

**Authors:** Krishna C. Suddala, Christine C. Lee, Paul Meraner, Mariana Marin, Ruben M. Markosyan, Tanay M. Desai, Fredric S. Cohen, Abraham L. Brass, Gregory B. Melikyan

**Affiliations:** 1 Department of Pediatrics, Emory University, Atlanta, GA, United States of America; 2 Department of Microbiology and Physiological Systems, University of Massachusetts Medical School, Worcester, MA, United States of America; 3 Rush University Medical Center, Department of Physiology and Biophysics, Chicago, IL, United States of America; 4 Gastroenterology Division, Department of Medicine, University of Massachusetts Medical School, Worcester, MA, United States of America; 5 Children’s Healthcare of Atlanta, Atlanta, GA, United States of America; Harvard Medical School, UNITED STATES

## Abstract

Late endosome-resident interferon-induced transmembrane protein 3 (IFITM3) inhibits fusion of diverse viruses, including Influenza A virus (IAV), by a poorly understood mechanism. Despite the broad antiviral activity of IFITM3, viruses like Lassa virus (LASV), are fully resistant to its inhibitory effects. It is currently unclear whether resistance arises from a highly efficient fusion machinery that is capable of overcoming IFITM3 restriction or the ability to enter from cellular sites devoid of this factor. Here, we constructed and validated a functional IFITM3 tagged with EGFP or other fluorescent proteins. This breakthrough allowed live cell imaging of virus co-trafficking and fusion with endosomal compartments in cells expressing fluorescent IFITM3. Three-color single virus and endosome tracking revealed that sensitive (IAV), but not resistant (LASV), viruses become trapped within IFITM3-positive endosomes where they underwent hemifusion but failed to release their content into the cytoplasm. IAV fusion with IFITM3-containing compartments could be rescued by amphotericin B treatment, which has been previously shown to antagonize the antiviral activity of this protein. By comparison, virtually all LASV particles trafficked and fused with endosomes lacking detectable levels of fluorescent IFITM3, implying that this virus escapes restriction by utilizing endocytic pathways that are distinct from the IAV entry pathways. The importance of virus uptake and transport pathways is further reinforced by the observation that LASV glycoprotein-mediated cell-cell fusion is inhibited by IFITM3 and other members of the IFITM family expressed in target cells. Together, our results strongly support a model according to which IFITM3 accumulation at the sites of virus fusion is a prerequisite for its antiviral activity and that this protein traps viral fusion at a hemifusion stage by preventing the formation of fusion pores. We conclude that the ability to utilize alternative endocytic pathways for entry confers IFITM3-resistance to otherwise sensitive viruses.

## Author summary

Expression of interferon-induced transmembrane proteins (IFITMs) in target cells potently inhibits fusion of many unrelated enveloped viruses, including the Influenza A virus, whereas arenaviruses, such as the Lassa fever virus, are resistant to these factors. The mechanism by which IFITMs interfere with the viral fusion step and the mechanism of virus escape from these restriction factors are poorly understood. Here, we tagged the late endosome-resident IFITM3 with fluorescent proteins and visualized single virus entry and fusion with endosomes in living cells expressing these constructs. Single virus and endosome tracking experiments demonstrate that the sensitive Influenza A virus is trapped within acidic IFITM3-positive endosomes that are not permissive for viral fusion. In contrast, the resistant Lassa virus consistently enters and fuses with endosomes lacking IFITM3. Our results imply that accumulation of IFITM3 in virus-carrying endosomes is a prerequisite for blocking fusion of diverse enveloped viruses and that viruses insensitive to this protein escape restriction by entering through distinct endosomal trafficking pathways that do not converge with IFITM3-positive compartments.

## Introduction

Fusion of enveloped viruses with the host cell membrane is a key step leading to infection. Viral fusion is initiated upon interactions between virus surface glycoproteins and cellular receptor(s) and/or upon the reduction in pH that follows endocytosis (reviewed in [1–3]). The extensive conformational changes that ensue in the viral glycoproteins promote fusion between viral and cellular membranes [4–6]. There is strong evidence that viral fusion—and membrane fusion in general—proceeds through a hemifusion intermediate defined as a merger of two contacting membrane leaflets without additional merger of distal leaflets that results in the formation of a fusion pore [6–8]. Accordingly, hemifusion is manifested as lipid mixing between viral and host membranes without viral content release, while full membrane fusion entails mixing of distinct aqueous contents delimited by the two membranes [4–6]. It has been demonstrated that sub-optimal conditions for membrane fusion including low density of viral glycoproteins, reduced temperature, and—where applicable—insufficiently acidic pH, favor dead-end hemifusion that does not progress to full fusion [9–12]. Thus, the progression to full viral fusion that culminates in the release of nucleocapsid into the cytoplasm is largely dependent on local conditions.

The viral envelope glycoproteins responsible for mediating membrane fusion are targets for neutralizing antibodies and virus entry inhibitors. In addition, new innate restriction factors inhibiting virus fusion have been discovered in recent years [13–15]. Among these factors is the family of small interferon-induced transmembrane proteins (IFITMs) that exhibits broad-range of antiviral activity [15–17]. This family includes IFITM1, which localizes predominantly at the plasma membrane, as well as IFITM2 and IFITM3, which contain an endocytic signal in their cytoplasmic N-terminal domain and thus localize to late endosomal and lysosomal membranes [18–21]. IFITMs effectively block entry of many unrelated enveloped viruses, including orthomyxoviruses (influenza A virus, IAV), paramyxoviruses (Respiratory Syncytial Virus, RSV), flaviviruses (Dengue, West Nile), filoviruses (Marburg, Ebola), and coronaviruses (SARS) [15–17, 20, 22–25]. IFITM3 alone is responsible for the bulk of antiviral effects of interferon in cell culture [15]. Importantly, mice lacking the *ifitm3* gene more readily succumb to IAV and RSV infection than control mice [26, 27]. There are, however, viruses that are resistant to IFITM-mediated restriction. Murine Leukemia Virus (MLV), Old and New World arenaviruses (Lassa Virus and Junin Virus, respectively), as well as several enveloped DNA viruses, are not affected by IFITMs [15, 28, 29].

The mechanism by which IFITMs inhibit fusion of most viruses, while sparing others, is not understood. We and others have shown that IFITM expression does not elevate the overall endosomal pH [15–19, 22, 30, 31] and, thus, should not block acid-triggered refolding of viral fusion proteins that initiate membrane fusion. Clues regarding the antiviral mechanisms of IFITMs come from their subcellular distribution which tend to correlate with IFITM’s potency against different viruses. IFITM2 and -3 better restrict viruses entering from late endosomes, while IFITM1 tends to be more effective against viruses that are thought to fuse with the plasma membrane or with early endosomes (reviewed in [17]). Indeed, expression of an IFITM3 mutant that redistributes the late endosome/lysosome-resident protein to the cell surface abolishes antiviral activity against IAV [32]. There are, however, exceptions to this rule. The fact that IFITM1 outperforms IFITM3 in restricting EBOV fusion [25] highlights the importance of cellular trafficking, as opposed to the steady state distribution, for antiviral activity. Also, a relatively weak IAV restriction exhibited by an IFITM1 chimera containing the N-terminal domain of IFITM3 that localizes to late endosomes suggests a role for other factors in addition to appropriate subcellular localization [21].

The most popular view of the mechanism of IFITM’s antiviral activity is that these proteins create “tough membranes” that are not conducive to fusion [17, 18, 22]. Two principal models for membrane stiffening by IFITMs have been proposed–a direct effect on the membrane in the immediate proximity of these proteins [19, 25, 33–35] that could involve changing the membrane fluidity and/or curvature [22, 33, 35], and an indirect effect through altering the lipid composition of endosomes [18]. Several lines of evidence support the proximity-based antiviral activity of IFITMs. First, as discussed above, there is a general correlation between the subcellular localization of IFITMs and their potency against viruses entering from distinct cellular compartments (reviewed in [17]). Second, IFITM3-mediated restriction, but not restriction by the plasma membrane-resident IFITM1, can be bypassed by forcing virus fusion with the plasma membrane [25, 30]. Third, IFITM incorporation into the viral membrane effectively inhibits fusion/infectivity [34, 36–38]. On the other hand, IFITM3 has been reported to bind to and inhibit the function of vesicle-associated membrane protein-associated protein A (VAPA) [18], the master regulator of endosome-ER lipid transport. While this model has been disputed by several groups [30, 35], a recent study provided evidence for the antiviral effect of cholesterol accumulation in late endosomes/lysosomes and confirmed accumulation of cholesterol in these compartments upon IFITM3 expression [39]. It thus remains unclear whether IFITMs must be present at the sites of virus fusion to block virus entry or affect fusion indirectly, by dysregulating lipid transport or metabolism.

We have previously shown that IFITM3 does not restrict the lipid-mixing (hemifusion) stage of viral fusion, but rather inhibits the formation of a fusion pore [30]. However, the inability to directly visualize IFITM3 in the context of virus entry into live cells precluded us from assessing whether this factor blocks fusion through a proximity-based mechanism. Here, we overcame this limitation by constructing a functional fluorescent IFITM3 protein and imaging virus co-trafficking and fusion with endosomal compartments in cells expressing this protein. Comparison of entry and fusion of IFITM3-sensitive (IAV) and–resistant (LASV) viruses by 3-color live cell imaging revealed that IAV enters into and remains trapped within endosomes enriched in fluorescent IFITM3, where viruses underwent hemifusion but failed to complete the fusion reaction. In contrast, LASV particles entered and fused with endosomes devoid of IFITM3, implying that LASV escapes restriction by utilizing an endocytic pathway distinct from that employed by IAV. Collectively, our results provide strong support to a proximity model by which presence of IFITM3 at the preferred sites of virus entry restricts viral fusion.

## Results

### Generation and characterization of a functional fluorescent IFITM3

**Fig 1:**
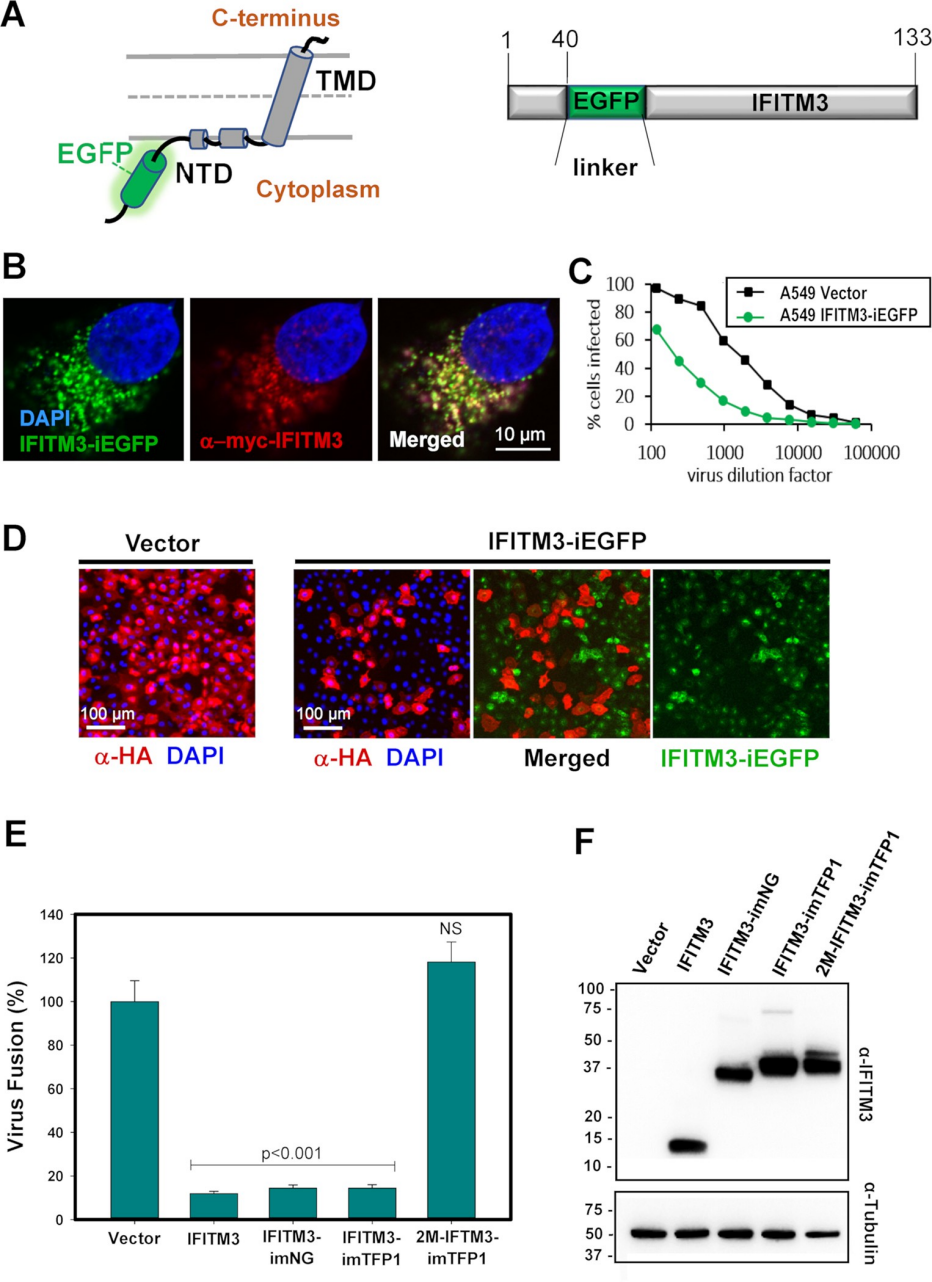
Construction and validation of IFITM3 tagged with florescent proteins. **(A)** Cartoon and schematic representation of IFITM3-iEGFP fusion protein. EGFP flanked with flexible linkers (GGGSGG) was inserted after residue 40 of IFITM3. NTD, N-terminal domain, TMD, transmembrane domain. **(B)** HeLa cells were transduced with both IFITM3-iEGFP (green) and N-terminally tagged myc-IFITM3 (red). Cells were fixed, permeabilized, immunostained with anti-myc tag antibody, and counterstained with DAPI. Co-localization of EGFP- and myc-tagged IFITM3 appears as yellow punctae. **(C)** IFITM3-iEGFP expressed in A549 cells inhibits viral infection of varied doses (dilutions of virus inoculum) of Influenza A/WSN/33 compared to an empty vector control. **(D)** A549 cells were transduced with empty vector (left) or IFITM3-iEGFP (three panels to the right), infected with influenza A/WSN/33, and immunostained 16 hours later for expression of HA antigen on the cell surface, which indicates productive virus infection. IFITM3-iEGFP expressing cells are protected from virus infection. **(E)** IAV pseudovirus fusion is inhibited in IFITM3 expressing A549 cells. HIV-1 pseudoviruses carrying BlaM-Vpr and IAV A/WSN/33 HA/NA were bound in the cold to A549 cells transduced with Vector, IFITM3, IFITM3-imNeonGreen (IFITM3-imNG), IFITM3-imTFP1 or the inactive mutant IFITM3 tagged with mTFP1, 2M-IFITM3-imTFP1. Fusion was initiated by shifting to 37°C and incubating for 2 hr. Data are means and error bars are SEM from three independent experiments performed in triplicate (**F**) Expression levels of different IFITM3 constructs in transduced A549 cells. Stable cell lines expressing an empty Vector, unlabeled IFITM3, IFITM3-imNG,IFITM3-imTFP1, or 2M-IFITM3-imTFP1 were lysed and analyzed by Western blotting using rabbit anti-IFITM3 and mouse anti-Tubulin antibodies as a loading control. (See also S1 Fig for cell fluorescence-based expression analysis).

To assess the co-distribution of viruses with IFITM3 at the time of fusion in live cells, we generated fluorescently-tagged IFITM3 protein. Linear N- or C-terminal fusions of EGFP (or similar fluorophores) with IFITMs render IFITMs nonfunctional. However, we found that the coding sequence of EGFP inserted into the N-terminal region of IFITM3, predicted to reside in the cytoplasm [40–42], generates a functional protein (Fig 1). First, to verify correct subcellular distribution of the fluorescent construct, we co-expressed IFITM3-iEGFP (iEGFP stands for “internal” EGFP) with an N-terminally myc-tagged IFITM3 in HeLa cells and confirmed their colocalization by fixing and immunostaining the cells (Fig 1B). Extensive colocalization between IFITM3-iEGFP and myc-IFITM3 suggests that subcellular localization of IFITM3 is not perturbed by incorporation of an EGFP tag. To test the functionality of the fluorescent construct, control A549 cells transduced with an empty vector (Vector) and cells transduced with IFITM3-iEGFP were infected with varied doses of influenza A/WSN/33 virus and the resulting infection measured by immunostaining cells for HA antigen. A549 cells were selected because they express very low endogenous levels of IFITM3 [15, 30, 31]. Cells expressing the EGFP-labeled IFITM3 were consistently much more resistant to influenza infection than control cells (Fig 1C). Microscopic analysis revealed that cells expressing intermediate to high levels of IFITM3-iEGFP did not stain for HA antigen (Fig 1D), demonstrating that IFITM3-iEGFP protects cells from IAV infection, similar to unlabeled IFITM3 [15, 30].

To facilitate multi-color live cell imaging of IFITM3 with fluorescently labeled viruses, we replaced the internal EGFP tag with cyan mTFP1 or bright green mNeonGreen protein. We next examined the ability of fluorescent IFITM3 constructs to inhibit IAV fusion using HIV-1 particles pseudotyped with influenza HA and NA proteins from the H1N1 A/WSN/33 strain (designated as IAVpp) and carrying the β-lactamase-Vpr (BlaM-Vpr) chimera, as described in [30]. A549 cells transduced with an empty vector, unlabeled IFITM3, IFITM3-imNG, or IFITM3-imTFP1 were inoculated with IAVpp, and the extent of viral fusion was measured after 2 h at 37°C based on the resulting cytosolic BlaM activity [43, 44]. Compared to the Vector control, IFITM3 severely restricts IAVpp fusion, as shown previously [30]. IFITM3-imNG and IFITM3-imTFP1 proteins were expressed in A549 cells at levels comparable to that of untagged IFITM3, as determined by Western blot analysis of respective cell lysates (Fig 1F), and also potently inhibited IAVpp fusion (Fig 1E). Comparable expression of the two fluorescent constructs is further supported by live cell fluorescence microscopy analysis (S1 Fig). Taken together, our results demonstrate both the appropriate subcellular localization and antiviral activity of fluorescently labeled IFITM3 constructs.

### IFITM3-containing endosomes are permissive for a lipid mixing stage of influenza virus fusion

**Fig 2:**
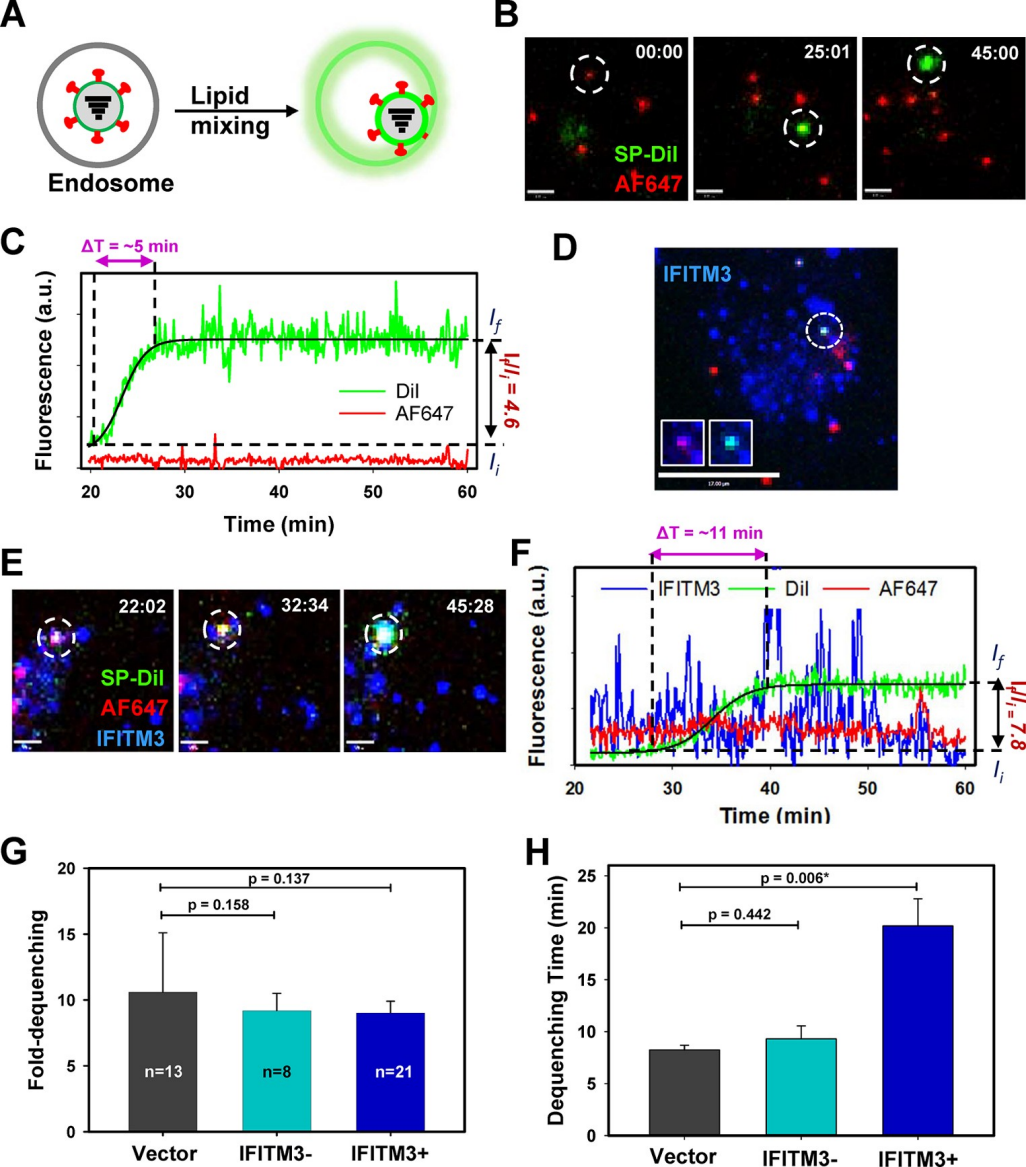
Fluorescent IFITM3 does not restrict IAV lipid mixing. **(A)** Schematic showing the single-virus hemifusion assay by monitoring dequenching of a lipophilic dye (SP-DiI_18_, colored green) resulting from lipid mixing between a virus and an endosome, yielding a green glow. HA was also fluorescently labeled (red), allowing it to be independently tracked. **(B)** Time series images show dequenching of SP-DiI_18_ upon entry of IAV co-labeled with AF647 (red) into A549 Vector cells. Scale bar 4 μm. **(C)** Fluorescence traces for the single IAV hemifusion event in (D) showing an increase in intensity of SP-DiI_18_ while the reference AF647 signal remains constant. The time for complete dequenching (Δt) and the dequenching ratio of final and initial mean intensities (*I*_*f*_/*I*_*i*_) are shown for this trace. (See S1 Movie.) **(D)** A549 cells stably transduced with IFITM3-imNG (IFITM3, blue) were monitored by time-lapse imaging, which shows SP-DiI_18_ dequenching as a result of lipid mixing between IAV and an endosome. Scale bar 17 μm. (See also S2 Fig) **(E)** Time series images show dequenching of SP-DiI_18_ upon entry of IAV co-labeled with AF647 with an endosome containing IFITM3-imTFP1. **(F)** Fluorescence traces for the single IAV lipid mixing event shown in (E). (See S3 Movie). **(G)** The average ratios of final (post-dequenching) and initial mean intensities (*I*_*f*_/*I*_*i*_) are shown from at least three independent experiments in A549 Vector and A549-IFITM3-imNG cells. Lipid mixing events were categorized based on whether they colocalized with IFITM3-imTFP1 puncta (IFITM3+) or occurred in the areas devoid of IFITM3-imNG signal (IFITM3-). Average ratios are plotted with standard errors. There are no significant differences in A549 Vector and IFITM3- average dequenching ratios or in A549 Vector and IFITM3+ endosomes. **(H)** The average dequenching times and standard errors are shown for A549 Vector, IFITM3- and IFITM3+ endosomes. The dequenching time between A549 Vector and IFITM3-negative endosomes was not significantly different, but lipid mixing was significantly slower in IFITM3+ endosomes.

Lipid mixing between two membranes is a necessary but not sufficient condition for complete fusion; lipids can diffuse through a hemifusion intermediate, without opening of a fusion pore (e.g., [11, 30, 45, 46]). We have previously shown that IFITM3 expression does not inhibit lipid mixing (hemifusion) between IAV and host endosomal membranes, but rather interferes with the formation of fusion pores [30]. The ability to visualize the distribution of functional fluorescent IFITM3 in living cells enables spatiotemporal analysis of viral fusion restriction. To test lipid mixing activity in the context of endosomes containing fluorescent IFITM3, we co-labeled infectious IAV (A/PR/8/34 H1N1) with a self-quenching concentration of the lipophilic dye SP-DiI_18_ and with the amine-reactive far-red Alexa Fluor647 dye (AF647) that labels surface glycoproteins of the virus [30]. As shown in the schematic diagram, single-virus hemifusion with an endosome can be detected by the appearance of bright SP-DiI_18_ spots resulting from dilution of this dye within an endosomal membrane (Fig 2A). Double-labeled IAV were incubated with cells at 4°C for 20 min, followed by the addition of pre-warmed Live Cell Imaging Buffer (LCIB), and imaging continued at 37°C. Representative snapshots from the time-lapse movie illustrate the single IAV SP-DiI_18_ (colored green) dequenching event around 25 min post-infection of Vector cells, indicating redistribution of the dye into an endosomal membrane (Fig 2B, S1 Movie). Fluorescent traces obtained by single IAV tracking show an increase in SP-DiI_18_ intensity over time, whereas the reference AF647 signal (red) remains relatively constant (Fig 2C). From these traces, the time required for complete dequenching (Δt) and the extent of dequenching (ratio of the initial and final mean intensities I_f_/I_i_) can be determined (Fig 2C).

To evaluate whether lipid mixing between IAV and IFITM3-containing compartments occurs, AF647 and SP-DiI_18_ labeled viruses were incubated with A549 cells expressing IFITM3-imNG, and virus entry/fusion monitored by three-color live cell microscopy (Fig 2D). As expected, fluorescent IFITM3 primarily localized to endosomes that exhibited retrograde and anterograde movement in living cells. The dynamics of IFITM3-imTFP1 intracellular transport is illustrated by S2 Movie. Snapshots of a single IAV show the hemifusion event that occurs within an IFITM3-imNG endosome (IFITM3), with the onset of SP-DiI_18_ dequenching detected at around 32 min (Fig 2E, S3 Movie). The overall kinetics of onset of lipid mixing was not affected by IFITM3 expression (S2A Fig). Fluorescence traces with levels of fluorescence intensity from IFITM3 (blue), SP-Dil_18_ (green), and AF647 (red) are shown with the time to SP-DiI dequenching (Δt) and dequenching ratio of the final and initial mean intensities (Fig 2F). It is clear that IAV HA-mediated lipid mixing is not inhibited by accumulation of IFITM3 in endosomes.

We next asked whether the presence of fluorescent IFITM3 affects the rate or extent of lipid exchange between IAV and the limiting membrane of a vesicle. The fold of SP-DiI_18_ dequenching was measured by calculating mean ratios of single IAV SP-DiI_18_ signals after and before dequenching (I_f_/I_i_) in A549 Vector cells and in A549-IFITM3-imNG cells occurring within IFITM3+ punctae (as in Fig 2F). The extent of SP-DiI_18_ dequenching was independent of virus colocalization with IFITM3-imNG endosomes at the time of lipid mixing (Fig 2G). Since the extent of dequenching of lipophilic dyes is proportional to their fold-dilution (e.g., [47, 48]), this result indicates that the average size of recipient endosomes is the same in control and IFITM3-expressing cells. However, the SP-DiI_18_ dequenching time (Δt) was significantly longer in IFITM3+ endosomes compared to Vector cells (Fig 2H), consistent with a hemifusion connection that is more restrictive for lipid diffusion in IFITM3+ endosomes compared to control cells. The slower redistribution of SP-DiI_18_ to endosomes enriched in IFITM3-imTFP1 is consistent with our previous conclusion that these events represent IAV hemifusion but not full fusion [30]. Indeed, lipid diffusion through both leaflets of a fusion pore is expected to be faster than diffusion through contacting leaflets of a hemifusion intermediate [49]. In another example, IAV SP-DiI_18_ dequenching can also occur with a bi-phasic increase in intensity, suggesting either transient fusion pore closure or representing the transition from a restrictive hemifusion structure that attenuates lipid diffusion to a fusion pore (S2 Fig).

### IFITM3 accumulates in influenza virus-carrying endosomes and blocks viral fusion

**Fig 3:**
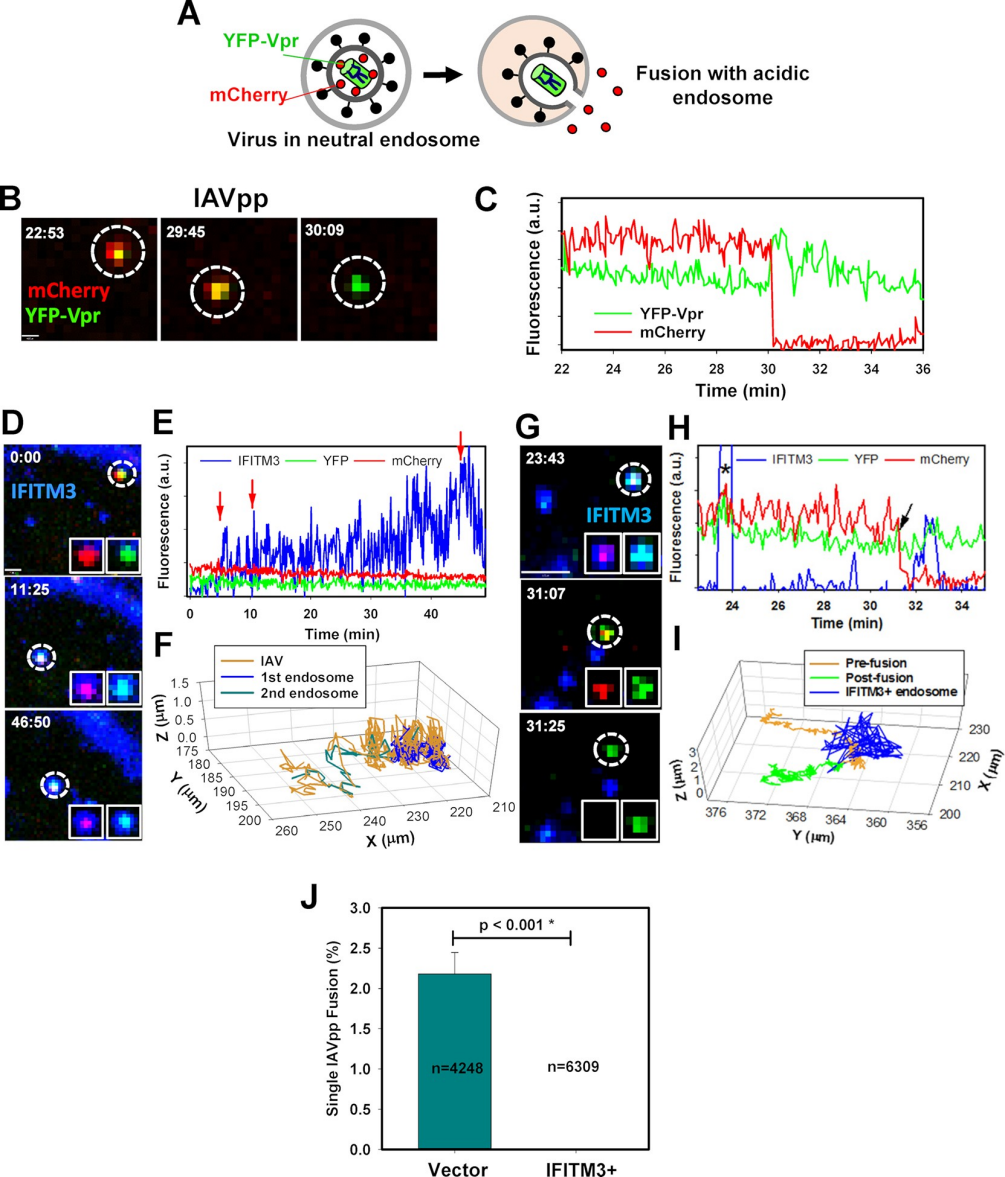
IFITM3 co-traffics with IAV and restricts viral fusion. **(A)** Schematic illustration of the single-virus fusion assay. Pseudoviruses bearing influenza virus HA and NA glycoproteins (IAVpp) are co-labeled with mCherry-2xCL-YFP-Vpr. Membrane fusion resulting in pore formation leads to the release of mCherry into the cytoplasm that appears as sudden loss of the red signal. In this model, the YFP marker remains associated with the core. **(B)** Timelapse images from the single IAV fusion event show the loss of mCherry signal at around 30 min, indicating a fusion event. Scale bar 2.8 μm. **(C)** Fluorescence trace of the fusion event shown in panel B. (See S4 Movie). **(D)** Time-lapse imaging was performed using IAVpp co-labeled with mCherry-2xCL-YFP-Vpr in A549-IFITM3-imTFP1 cells. Single frames show IAVpp co-trafficking within an IFITM3+ compartment without undergoing fusion. Scale bar 0.8 μm. **(E)** Fluorescence intensity analysis of the virion shown in panel D. The red arrow around 7 minutes indicates virus entry into an IFITM3+ endosome and the second and third arrows mark subsequent merger of the virus-carrying endosome with additional IFITM3+ vesicles around 10 min and 43 min, leading to increases in the IFITM3 intensity. **(F)** Particle tracking in 3D confirms co-trafficking of the IAVpp (dark yellow) with an IFITM3+ endosome (green) followed by merging with a second IFITM3+ endosome (blue). (See S5 Movie). **(G)** IAVpp were labeled as in A. Time series images show fusion of IAVpp within an IFITM3 expressing cell at a location that does not contain above-threshold amounts of IFITM3-imTFP1. IAVpp comes into proximity with an IFITM3-containing compartment but does not co-traffic with it. Fusion occurs prior to transient colocalization with an IFITM3-imTFP1 spot. (See also S3 Fig). **(H)** Fluorescence traces of the particle shown in (G). Transient colocalization with an IFITM3+ endosome and subsequent fusion are marked with an asterisk and an arrow, respectively. (See S6 Movie). **(I)** 3D trajectories of IAVpp and an endosome shown in panel G confirm that, prior to fusion (yellow trace), the virus-carrying endosome only transiently encounters the IFITM3+ endosome (blue). Neither before (dark yellow trace) nor after fusion (green trace) of the particle co-trafficking with the IFITM3+ endosome was observed. **(J)** Single IAVpp fusion efficiency in A459 Vector and IFITM3-imTFP1 (IFITM3+) cells.

To visualize single IAV fusion in A549 cells, we pseudotyped the HIV-1 core with H1N1 HA and NA glycoproteins, as previously described [30]. IAVpp was labeled with a bi-functional mCherry-2xCL-YFP-Vpr construct, with a 2xCL tandem cleavage site for the viral protease that is cleaved during virus maturation, generating a free mCherry and a core-associated YFP-Vpr [50]. Virus fusion is detected based upon the release of mCherry into the cytoplasm through a fusion pore, while the YFP-Vpr marker remains in the viral core (Fig 3A). Labeled IAVpp were bound to A549 Vector cells in the cold by spinoculation (see Methods). Virus fusion was synchronously initiated by adding pre-warmed LCIB and visualized by two-color live cell microscopy for 2 hours with mCherry and YFP signals acquired every 6 seconds. Single IAVpp entered and fused with A549 cells, in agreement with our published data [30]. Time series images show the initial trafficking of a representative mCherry/YFP-Vpr labeled virus until fusion occurs, as evidenced by the loss of mCherry fluorescence, while the YFP-Vpr signal remains relatively constant (Fig 3B and 3C, S4 Movie).

Considering that the overwhelming majority of the IAV lipid mixing events occur in IFITM3-containing endosomes (Fig 2) and based upon our previous observation that IFITM3 expression inhibits single IAVpp fusion [30], we hypothesized that a sufficiently high local IFITM3 concentration is required for restriction of IAV fusion. To test this hypothesis, we synchronized IAVpp entry into A549-IFITM3-imTFP1 cells, as described above, and monitored mCherry/YFP-Vpr-labeled viral particles using three-color live cell imaging. Time series images show the IAVpp entering the IFITM3+ compartment and co-trafficking without undergoing fusion (Fig 3D). The fluorescence traces obtained by single virus tracking confirm entry (indicated by the red arrow) into an IFITM3+ endosome. At a later time, the virus-carrying endosome encounters and merges with another IFITM3+ compartment, as indicated by the second red arrow around 36 min (Fig 3E, S5 Movie). This pseudovirus did not fuse (release mCherry) for as long as the time-lapse imaging was performed. Co-trafficking of IAVpp with an IFITM3+ compartment can be visualized by examining 3D trajectories of the virus and relevant endosomes, which shows IAVpp co-trafficking with the first and then the second endosomal compartments containing IFITM3-imTFP1 (Fig 3F). Importantly, analysis of 6309 particles did not reveal a single viral fusion event occurring after extensive IAVpp co-trafficking with an IFITM3+ compartment.

In addition to tracking IAV particles that co-traffic with IFITM3+ compartments for an extended period of time, transient encounters with IFITM3+ compartments that did not inhibit subsequent IAVpp fusion were also observed. An example trace of an IAV pseudovirus shows a brief (~40 sec) apparent co-localization with an IFITM3+ compartment at around 23 minutes (Fig 3G and 3H, S6 Movie). Examination of 3D trajectories demonstrate that the particle does not significantly colocalize/co-traffic with the IFITM3+ endosome and that fusion occurs at around 31 minutes with no above-background IFITM3-imTFP1 signal (Fig 3H and 3I). Another example of false colocalization of IAVpp with an IFITM3+ endosome is shown in S3 Fig and S7 Movie. The viral particle appears to transiently colocalize with an IFITM3+ endosome when visualized in 2D, but particle tracking performed in 3D shows that the viral particle and the IFITM3+ endosome traffic in different Z-planes. These observations suggest that a transient and chance encounter of a virus carrying endosome with an IFITM3+ endosome is not sufficient to restrict fusion. In contrast, fusion is blocked after sustained and prolonged IAVpp co-trafficking with an IFITM3+ endosome, which strongly implies that the virus is being carried by an IFITM3-enriched compartment.

Further analysis of time-lapse acquisitions performed in at least 15 independent experiments shows that, on average, 2.2% of IAVpp fuse in Vector cells and none of the 6309 analyzed particles in IFITM3+ endosomes underwent fusion (Fig 3J). Our results thus demonstrate, for the first time, that the presence of IFITM3 in the endosomes carrying the virus is key to restriction of IAV fusion. The unimpeded lipid mixing between IAV and IFITM3+ endosomes, together with the lack of viral content release, strongly imply that IFITM3 traps the IAV fusion at a hemifusion stage by blocking the formation of a small fusion pore (in agreement with our previous study [30]).

### Conditions that render IFITM3 inactive rescue IAV fusion with IFITM3-containing compartments

Inhibition of IAVpp fusion after sustained co-trafficking with IFITM3-imTFP1-enriched endosomes may occur through a direct block of viral fusion by the restriction factor. Alternatively, IFITM3 may indirectly interfere with IAV fusion by altering the properties of endosomes, such as the luminal pH. The overall acidity of IFITM3+ endosomes was assessed by loading A549-IFITM3-imTFP1 cells with the acidic compartment marker, LysoTracker Red (S4 Fig). Imaging of fixed cells shows strong cytoplasmic colocalization between IFITM3+ endosomes and LysoTracker Red positive compartments, with only a small fraction of peripheral IFITM3+ endosomes lacking a detectable LysoTracker Red signal (S4A Fig, *Inset*). Analysis of multiple fields of view confirms that most IFITM3+ endosomes, with the exception of a small number of peripheral endosomes, accumulate the lysosomal marker (S4B Fig). This finding is consistent with progressive acidification of early IFITM3-containing endosomes through a maturation process and thus supports the notion that virus-carrying late IFITM3+ endosomes are acidic.

**Fig 4:**
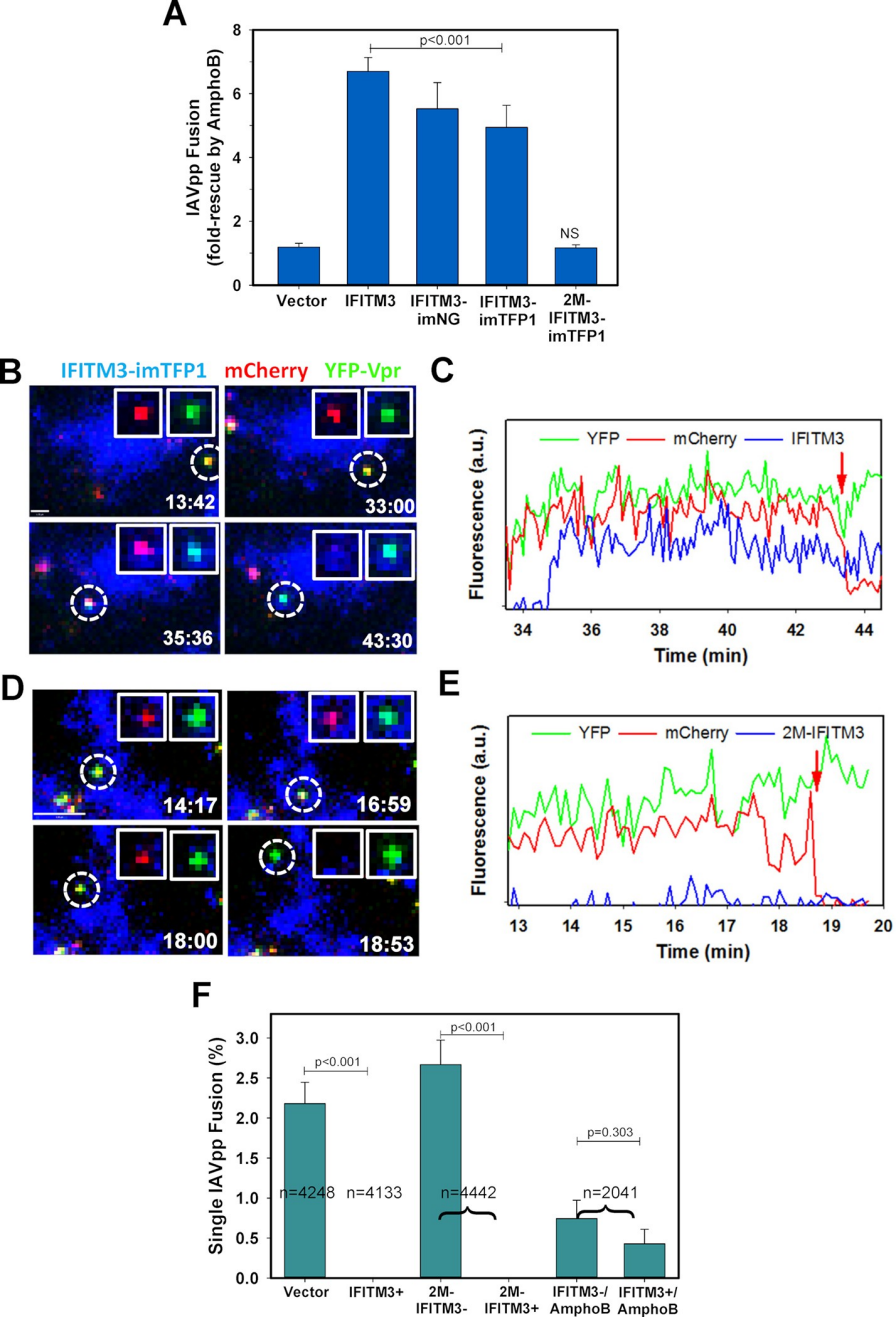
Amphotericin B treatment allows IAV fusion with IFITM3-containing endosomes. **(A)** AmphoB rescues IAVpp fusion with IFITM3 expressing cells. IAVpp carrying BlaM-Vpr were used to infect A549 Vector, IFITM3, IFITM3-imNeonGreen (IFITM3-imNG), IFITM3-imTFP1, or 2M-IFITM3-imTFP1 cells in the presence of 1 μM AmphoB and viral fusion was measured by the BlaM assay. Data are fold-increase compared to fusion in the absence of AmphoB and SEM from 2 independent triplicate experiments. Lack of significant AmphoB effect on IAVpp fusion with unlabeled IFITM3 versus 2M-IFITM3-imTFP1 expressing cells is associated with their relatively high permissiveness for IAV fusion (see Fig 1F). **(B)** IAVpp carrying mCherry-2xCL-YFP-Vpr were imaged with A549-IFITM3-imTFP1 cells treated with 1 μM AmphoB. Time-lapse images show the viral particle entering an IFITM3+ compartment around 35 minutes and co-trafficking with IFITM3-imTFP1 until fusion occurs around 43 minutes. Scale bar 1.3 μm. **(C)** A fluorescence trace corresponding to the fusion event in panel C in the presence of AmphoB. (See S8 Movie). **(D)** Double-labeled IAVpp were pre-bound to 2M-IFITM3-imTFP1 cells as in (B). Images show IAVpp in an IFITM3+ cell that does not co-traffic with IFITM3+ compartments. Scale bar 5.9 μm. **(E)** Fluorescence traces for a single particle fusion in 2M-IFITM3-imTFP1 cells corresponding to the particle in panel E. (See S9 Movie). **(F)** Single IAVpp fusion efficiency in A459 Vector, IFITM3-imTFP1, 2M-IFITM3-imTFP1, and IFITM3-imTFP1 cells treated with 1 μM AmphoB; means are plotted with SEM from at least 11 independent experiments. IFITM3- and IFITM3+ denotes fusion with IFITM3-negative and -positive endosomes, respectively. (See also S5 Fig).

To further test whether IFITM3+ compartments are otherwise permissive for viral fusion, we sought to render IFITM3-imTFP1 inactive by pretreating cells with Amphotericin B (AmphoB), which is known to antagonize the antiviral activity of IFITM3 ([35] and Fig 4A). We also used the inactive oligomerization-defective IFITM3 mutant, with alanine substitutions at F75 and F78 (denoted 2M) [21]. As expected, the 2M-IFITM3-imTFP1 mutant did not inhibit IAVpp fusion (Fig 1E), in spite of being expressed at a level comparable to IFITM3 and IFITM3-imTFP1 (Fig 1F).

We next probed the ability of single IAVpp to fuse with IFITM3+ compartments under conditions that rescue bulk IAVpp fusion. A549-IFITM3-imTFP1 cells were infected with IAVpp labeled with mCherry-2xCL-YFP-Vpr, as above, in the presence of 1 μM AmphoB. As seen with a bulk fusion assay (Fig 4A), single IAVpp fuses with IFITM3+ endosomes in the presence of AmphoB. Representative single virus images show the entry and subsequent co-trafficking of an IAV particle with an IFITM3+ compartment and fusion within the compartment, as indicated by the sudden loss of mCherry (Fig 4B and 4C, S8 Movie). Of the 23 total fusion events that occur in IFITM3-imTFP1 cells treated with AmphoB, 7 particles co-traffic and fuse with IFITM3+ compartments (Fig 4G). In contrast to IFITM3-imTFP1 expressing cells in the presence of AmphoB, IAVpp exclusively fused at sites devoid of the mutant IFITM3 in 2M-IFITM3-imTFP1 cells (Fig 4D and 4E, S9 Movie). None of the IAVpp fusion events of the total 4442 particles annotated in 2M-IFITM3 cells co-traffic with IFITM3+ compartments. Fig 4D and 4E illustrates this phenomenon, whereby an IAVpp particle fuses within a 2M-IFITM3-imTFP1 cell but does not co-traffic with appreciable local 2M-imTFP1 maxima. These results suggest that loss of antiviral activity of the F75/78A IFITM3 mutant may be due to its altered subcellular distribution that prevents co-trafficking with IAV. This is in contrast to AmphoB, which renders wild-type IFITM3-imTFP1 inactive without affecting its trafficking pathways. Of note, both conditions that rescued the IAVpp fusion delayed the fusion kinetics relative to untreated cells expressing IFITM3-imTFP1 (S5 Fig), indicating a global effect on the rate of virus endocytosis and entry into permissive compartments. Together, the above results support the notion that IFITM3 inhibits IAV fusion through a proximity-based mechanism–by co-trafficking with the virus and accumulating in compartments that are otherwise permissive for IAV fusion.

### LASV fusion in A549 cells proceeds through a viral membrane permeabilization step

**Fig 5:**
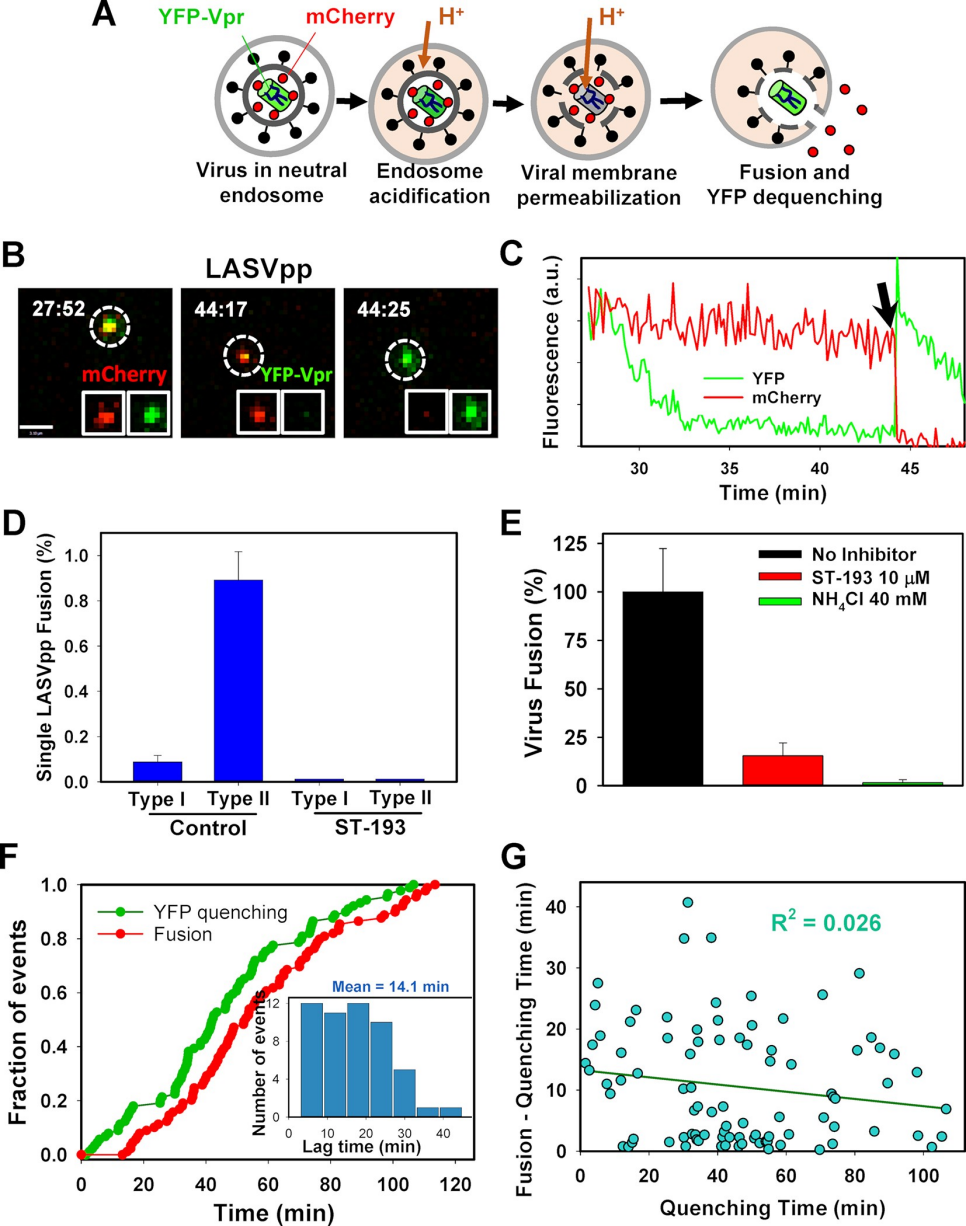
LASV pseudovirus fusion with A549 cells is preceded by a virus membrane permeabilization step. **(A)** Illustration of the fusion event depicting different stages of LASV fusion with an endosome. The YFP signal is quenched in an acidic endosome, due to viral membrane permeabilization, prior to fusion. Fusion is manifested in the loss of the mCherry signal with the concomitant recovery of the YFP-Vpr signal resulting from re-neutralization of the virus interior. **(B)** Time-lapse images of single LASVpp showing YFP quenching followed by fusion (dequenching of YFP signal and loss of mCherry). Scale bar 3.1 μm. **(C) F**luorescence traces of LASVpp fusion in an A549 Vector cell are shown for panel B. Fusion occurs at about 44 min, as seen by an instantaneous dequenching of YFP fluorescence and concomitant loss of the mCherry signal. (See S10 Movie). **(D)** A549 Vector cells were treated with 10 μM ST-193 or left untreated and the extent of single LASVpp fusion was measured. **(E)** The effect of ST-193 (10 μM) on LASVpp fusion was tested using the BlaM assay in A549 Vector cells. In control wells, infection was performed in the presence of 40 mM NH_4_Cl. Data points are mean and STD from 2 independent experiments performed in duplicates. **(F)** Kinetics of single LASVpp YFP quenching and fusion (loss of mCherry) events in A549 Vector cells. *Inset*: Distribution of the lag time between YFP quenching and fusion for each viral particle. **(G)** Correlation between the lag time between YFP quenching and fusion and the waiting time for YFP quenching for each fusion event. Solid line is linear regression.

To visualize single LASV entry and fusion, which is not restricted by IFITM3 in A549 cells [15], we pseudotyped the HIV-1 core containing the bi-functional mCherry-2xCL-YFP-Vpr marker with the LASV GPc envelope glycoprotein complex to generate LASV pseudoparticles (LASVpp). LASVpp imaging in A549 cells confirmed the ability to track single particles and detect their fusion (release of mCherry) in late endosomal compartments (Fig 5B, S10 Movie). Interestingly, LASVpp fusion exhibited a unique feature rarely seen for other viruses, including IAV. The YFP-Vpr fluorescence, which is markedly decreased at mildly acidic pH [51, 52], was consistently quenched at some point prior to viral fusion, demonstrating acidification of intraviral pH [53, 54] (schematized in Fig 5A). Single frame images show that YFP-Vpr signal quenched for ~10 min before viral fusion, which is observed as the loss of mCherry signal (red) and concomitant reappearance of the YFP-Vpr signal (Fig 5B and 5C, arrow). The dequenching of YFP fluorescence can be attributed to the re-neutralization of the virus’ interior through a fusion pore connecting it to the cytoplasm [52, 54, 55]. Based on the differences in IAVpp and LASVpp fusion, we classified single virus fusion events into “Type I”, in which mCherry signal is lost without acidification of the virus interior (YFP quenching), as observed in IAV fusion, and “Type II” events, in which acidification of the virus interior occurs prior to fusion (mCherry release), as observed in LASV fusion. In A549 cells, only 9% of LASVpp fusion events are Type I, while most particles—91%—undergo Type II fusion (Fig 5D). Of note, YFP-Vpr quenching occurs for most particles not undergoing fusion at later times after infection due to a non-specific acidification of the viral interior in late acidic compartments. These events representing a non-productive entry of LASVpp were excluded from analysis.

Additional experiments to confirm single LASVpp fusion in A549 cells were also performed. Control cells were treated with a broad-spectrum arenavirus entry inhibitor, ST-193 [56], which abrogated single LASVpp fusion events (Fig 5D). A total of 90 Type II events were observed in at least 24 independent experiments, with 6430 viral particles in Vector control cells and 5264 viral particles observed in cells treated with ST-193. LASVpp fusion with A549 cells measured by a bulk BlaM assay also demonstrated potent inhibition of viral fusion in the presence of 10 μM ST-193 or upon raising the endosomal pH by 40 mM NH_4_Cl (Fig 5E). Thus, the observed changes in fluorescent signals faithfully represent single LASVpp fusion.

Consistent with the lag between YFP quenching and fusion (Fig 5B and 5C), the kinetics of LASVpp fusion lagged behind the YFP quenching events (Fig 5F). The average lag time between YFP-Vpr quenching and fusion for LASVpp in A549 cells is 14.1 minutes (Fig 5F, inset). To test if the observed lag was due to the requirement for further virus trafficking to fusion-permissive compartments, we asked if it depended on how long a virus trafficked prior to YFP-quenching (Fig 5G). The lag between quenching and fusion does not appear to be correlated with the waiting time for quenching (R^2^ = 0.0263), suggesting that LASVpp fusion following the YFP quenching is a stochastic event that does not depend on the virus trafficking history. Although acidification of the virus interior does not directly report the time of acidification of endosomal lumen, the above results demonstrate that LASV GPc retains fusion-competence under acidic conditions for a considerable time before it fuses with permissive late endosomes, perhaps after binding to LAMP1 [57, 58].

### LASV traffics into and fuses with IFITM3-negative compartments

**Fig 6:**
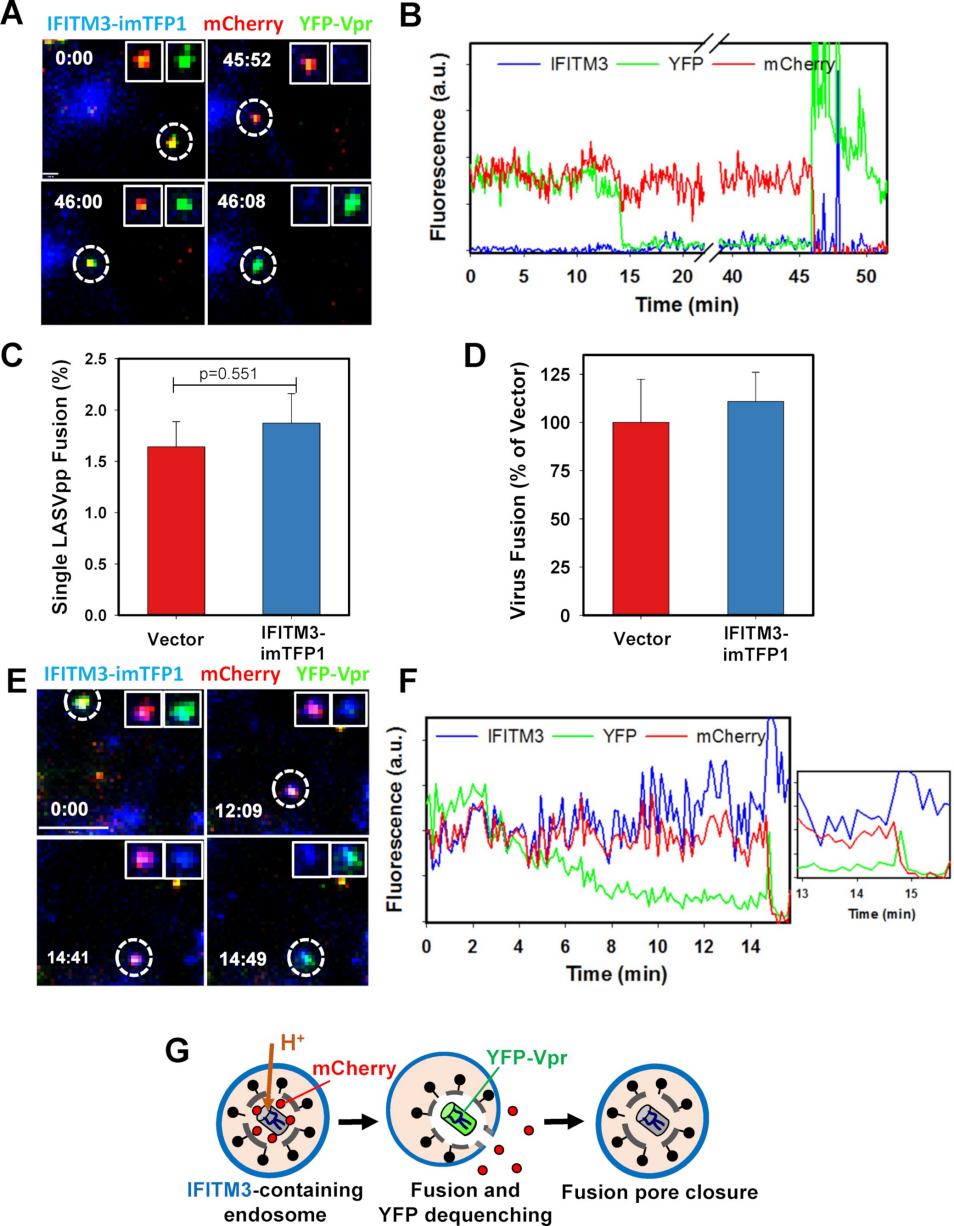
LASV pseudoviruses fuse with endosomes lacking IFITM3. LASVpp labeled with mCherry-2xCL-YFP-Vpr were allowed to enter and fuse with A549-IFITM3-imTFP1 cells. **(A)** Images of a single LASVpp fusion just prior to transient colocalization with an IFITM3-imTFP1-containing vesicle. Scale bar 1.9 μm. **(B)** Fluorescence traces of the LASVpp fusion event shown in panel A. (See S11 Movie). **(C)** Analysis of >6 independent live cell imaging experiments shows that, on average, LASVpp fuse in A549 Vector and A549-IFITM3-imTFP1 cells with similar efficiency. (**D**) IAVpp fusion efficiency with A549 Vector and A549-IFITM3-imTFP1 cells was measured by the BlaM assay. Data are means and SEM from 3 independent triplicate experiments. **(E)** Single LASVpp images showing rare mCherry release from within an IFITM3 containing vesicle. This release is associated with an unusual transient dequenching of the YFP signal. The IFITM3+ vesicle can be seen in the same location in the image with no YFP signal. Scale bar 8.0 μm. **(F)** Trace of the LASV particle in (C) showing that the virus co-traffics with the IFITM3+ vesicle before fusion. *Inset*: Transient dequenching of YFP signal at the time of mCherry release. (See S12 Movie). **(G)** A schematic depicts the transient fusion event from (E) and (F). Re-quenching of the YFP signal is consistent with pore closure and re-acidification of the virus interior. (See also S6 Fig).

We next assessed the basis for LASV resistance to IFITM3 restriction. A549-IFITM3-imTFP1 cells were infected with LASVpp labeled with mCherry-2xCL-YFP-Vpr, as above. Single particle imaging revealed that LASVpp did not co-traffic with IFITM3-imTFP1-positive endosomes and that subsequent viral fusion occurred at sites devoid of this fluorescent restriction factor (Fig 6A, S11 Movie). Analysis of single LASVpp fluorescence intensities in A549-IFITM3-imTFP1 cells shows a typical Type II fusion event which occurs within an endosome lacking above-background amounts of IFITM3-imTFP1 (Fig 6B).

Live cell imaging experiments were performed at least 6 times independently, and on average, 1.64% and 1.87% of double-labeled LASVpp particles bound to cells fused in A549 Vector and A549-IFITM3-imTFP1 cells, respectively (p = 0.551) (Fig 6C). These data confirm previous reports that the expression of IFITM3 does not affect LASV fusion [15, 30]. In addition, the kinetics of LASVpp fusion in control and A549-IFITM3-imTFP1 cells were not significantly different (S6A Fig). LASVpp fusion kinetics were the same regardless of IFITM3-imTFP1 expression, as observed using the BlaM assay and stopping fusion at varied times by NH_4_Cl addition (S6B Fig).

We note that in one or two rare examples, LASVpp fusion appears to occur in an endosome containing detectable IFITM3-imTFP1 signal. Representative images and fluorescence traces show co-trafficking of a LASV particle within an IFITM3+ endosome until fusion occurs around 14 min post-infection (Fig 6E and 6F and *Inset*, S12 Movie). This unique event is atypical of the majority of tracked particles due to several reasons: (1) there is an apparent colocalization with IFITM3+ beginning at time 0; and (2) LASVpp rarely fuse as early as 14 min post-infection. Most importantly, the fusion event in Fig 6E and 6F appears to represent transient fusion pore opening, as indicated by the sudden re-quenching of YFP, or re-acidification of the virus interior following pore closure (Fig 6E–6G). We report this instance to illustrate that, while LASVpp typically avoids IFITM3+ endosomes, miniscule levels of fusion may occur within IFITM3+ compartments. Overall, LASVpp exhibit a strong tendency to bypass IFITM3+ endosomes and this important feature likely represents the mechanism by which this virus escapes restriction.

### IAV but not LASV particles increasingly colocalize with IFITM3-containing vesicles over time

**Fig 7:**
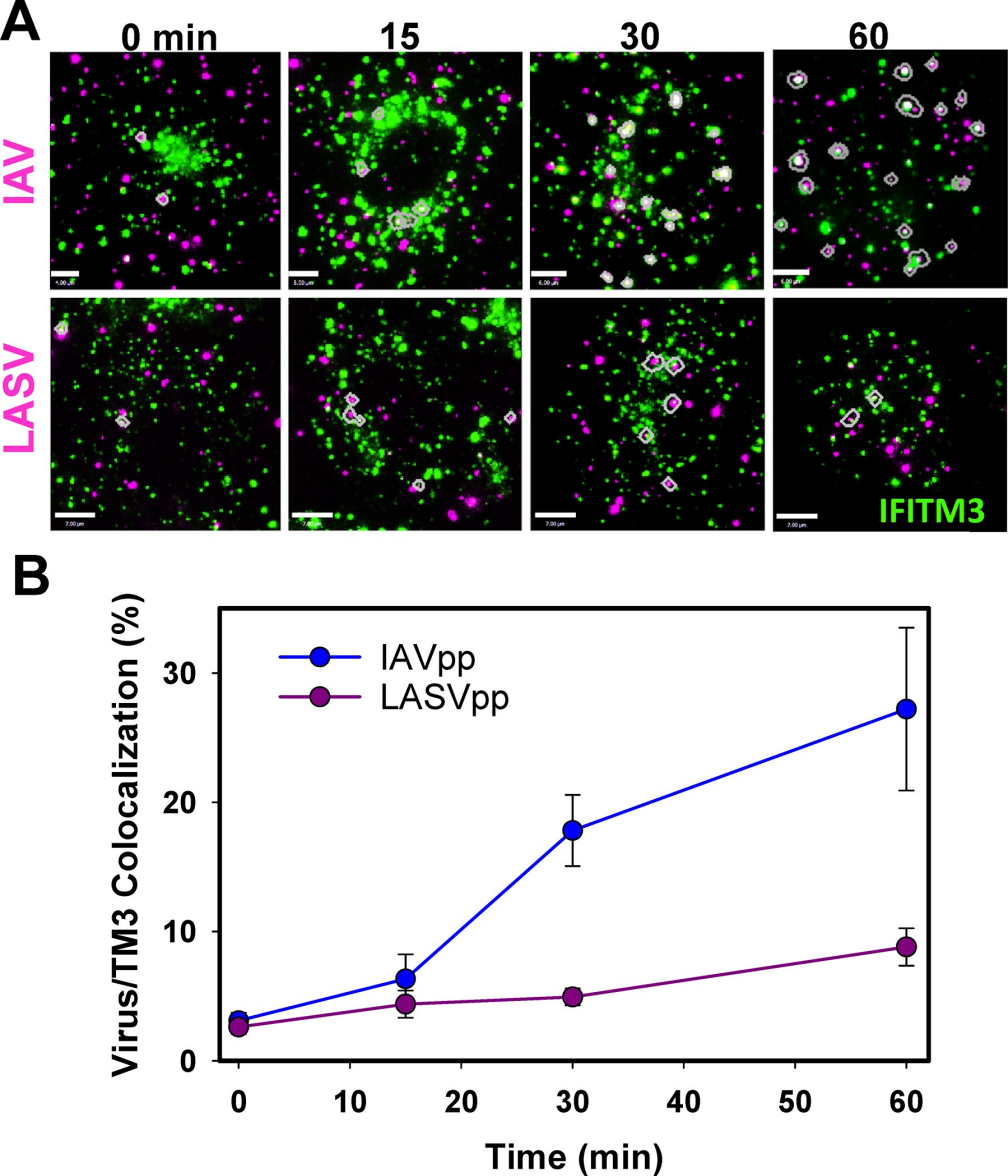
IAV but not LASV particles colocalize with IFITTM3-containing vesicles. A549 cells expressing IFITM3-imNG were incubated in the cold for 1.5 hr with particles pseudotyped with IAV HA/NA or LASV GPc and labeled with HIV-1 Gag-mCherry (red). Cells were washed to remove unbound viruses, incubated at 37°C for varied times, fixed in 4% paraformaldehyde and imaged. **(A)** Representative images at different time points show IAVpp (top) and LASVpp (bottom) co-localization with IFITM3-imNG (green) containing vesicles in A549 cells. The colocalized particles are circled in magenta. Scale bar 3 μm (top and bottom). **(B)** Quantification of IAVpp and LASVpp colocalization with IFITM3-imNG endosomes over time. Data at each time point are mean ± SEM from at least 5 different fields of view containing multiple cells.

Analysis of single IAVpp and LASVpp co-trafficking with IFITM3-imTFP1 endosomes in the context of fusion (Figs 3–6) suggests that IAVpp would be trapped in IFITM3-positive endosomes/multivesicular bodies, while LASVpp would not. To test this notion, we followed the bulk virus uptake and transport in live A549 cells expressing IFITM3-imNG. Cells were incubated in the cold for 1.5 hr with IAVpp or LASVpp labeled with an internal fluorescent marker, Gag-mCherry, to allow virus binding. Cells were then incubated for indicated times (0, 15, 30, and 60 min) at 37°C, fixed and imaged at high spatial resolution. Representative images of IAVpp and LASVpp co-localization with IFITM3 containing vesicles at different time points are shown in Fig 7A. Quantification of virus colocalization with IFITM3-imNG over time shows that IAVpp increasingly co-localizes with IFITM3-imNG compartments, while LASVpp does not show a significant increase in colocalization up to 1 hr post-infection (Fig 7B). These data support the hypothesis that restriction-sensitive viruses (as is the case for IAV) co-traffic with IFITM3, while resistant viruses (like LASV) are transported through distinct endosomal compartments devoid of this restriction factor.

### IFITM3 incorporation into the viral membrane inhibits fusion mediated by both IFITM3-sentitive and–resistant viral glycoproteins

**Fig 8:**
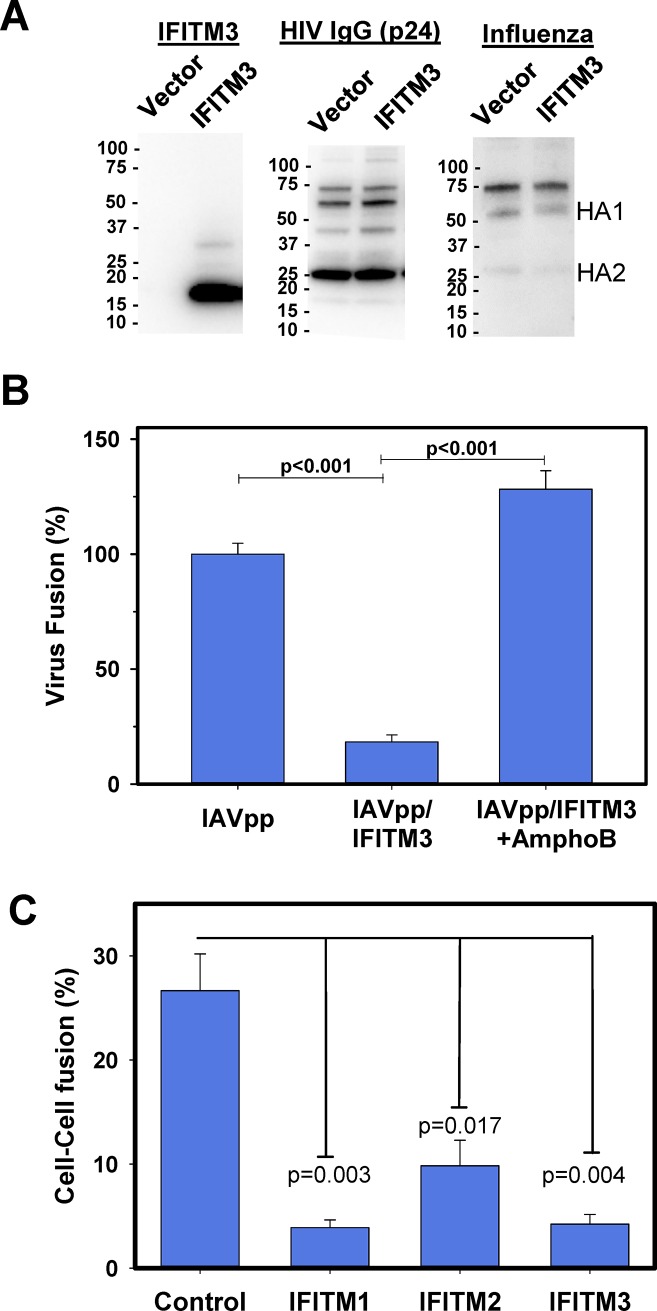
IFITM3 proximity to viral fusion proteins allows virus restriction. **(A-D)** IFITM3 incorporates into virions and restricts viral fusion in A549 cells. **(A)** Western blot of control and IFITM3-containing IAVpp prepared through co-transfection of 293T/17 cells with additional plasmids–either empty vector (Vector) or IFITM3 (IFITM3), respectively. Blots were probed with anti-IFITM3, anti-p24, and anti-influenza HA antibodies. **(B)** IAVpp containing BlaM-Vpr and lacking or containing IFITM3 in the presence or absence of AmphoB were used to infect A549 cells and the resulting viral fusion measured by the BlaM assay. Data are means and SEM based on three experiments performed in triplicate (except for IAVpp/IFITM3 treated with AmphoB, which was performed twice in triplicate). (**C**) LASV GPc induced cell-cell fusion is inhibited by IFITMs. Co-cultures of 293T cells stably expressing IFITM1, IFITM2 or IFITM3 with Cos7 transiently expressing LASV GPc were co-cultured for 30 min at room temperature and exposed to a pH 5.0 citrate buffer for 20 min at 37°C to induce LASV fusion. The extend of fusion was measured by microscopy-based dye transfer assay, as described in Materials and Methods. Data are mean with SEM from 3 independent experiments. (See also S6 Fig).

To further test whether the presence of IFITM3 is necessary and sufficient to restrict IAV fusion, we generated control IAV particles that incorporated IFITM3 through co-expression in virus-producing cells. These pseudoviruses contained BlaM-Vpr to assess their fusion-competence. IFITM3 incorporation into virions and its possible effects on HIV-1 maturation or the influenza HA incorporation into pseudoviruses were verified by Western blotting (Fig 8A). IFITM3 was present in pseudoviruses prepared in producer cells expressing IFITM3 but not in the Vector control. Furthermore, the amount of p24 protein and influenza HA were the same in the two preparations (Fig 8A), indicating that IFITM3 incorporation does not perturb the expression or proteolytic processing of HA.

To probe the fusion activity of IFITM3-containing pseudoviruses, A549 cells were incubated with IAVpp/IFITM3 (or control viruses lacking IFITM3) at 4°C for 30 min, followed by incubation for 2 hours at 37°C in either drug-free medium or medium supplemented with AmphoB (which rescues IAV fusion in A549-IFITM3-imTFP1 cells, Fig 4A and [35]). Compared to the control IAVpp, fusion of IAVpp containing IFITM3 was potently inhibited (p<0.001, Fig 8B). Interestingly, AmphoB rescued IAVpp/IFITM3 fusion (Fig 8B), suggesting a direct effect of this antibiotic on IFITM3 or the viral membrane that is independent of cellular processes, including endocytic transport. The diminished ability of IAVpp produced in the presence of IFITM3 to fuse with target cells was not caused by IFITM3-containing extracellular vesicles present in the viral preparations, as have been suggested in [59]. Viral fusion was not significantly diminished when A549 cells were infected with a mixture of control IAVpp and extracellular medium from cells transfected only with an IFITM3-expressing plasmid (S7A Fig). Although IFITM3-containig extracellular vesicles were effectively concentrated by our virus concentration protocol using LentiX (S7B Fig), these vesicles did not modulate the fusion activity of IAVpp under our experimental conditions involving a brief exposure of target cells to the virus and vesicles. Taken together, these results suggest that the presence of IFITM3 is necessary and sufficient to restrict IAV fusion, irrespective of whether or not IFITM3 is expressed in the target or viral membrane.

Further evidence supporting the proximity-based antiviral activity of IFITM3 was obtained by measuring LASV GPc-mediated cell-cell fusion. In this model, cell fusion is triggered by exposure to low pH, which bypasses the need for endocytic trafficking that may sort LASVpp away from IFITM3+ compartments. Cos7 cells transiently expressing LASV GPc were brought in contact with 293T cells stably expressing IFITM1, IFITM2 or IFITM3 [22] or, in control experiments, with parental 293T cells. Cos7 and 293T cells were pre-loaded with different cytosolic fluorescent dyes to monitor fusion initiated by an acidic buffer, as previously described [22]. In stark contrast to LASVpp fusion with IFITM3-expressing cells (Fig 6), GPc-mediated cell-cell fusion was markedly inhibited by all three IFTIMs expressed in target cells (Fig 8C). This result supports the notion that LASV GPc is not inherently resistant to IFITM restriction and that the reason LASV is insensitive to IFITM3 expression is through its usage of trafficking pathways that are distinct from those used by IFITM3.

## Discussion

A remarkable breadth of enveloped viruses that are restricted by IFITM proteins suggests a universal mechanism for antiviral activity that likely involves altering the properties of the host cell membranes in a way that precludes viral fusion. It remains unknown how IFITMs exert their antiviral effects and, equally importantly, how arenaviruses and MLV escape restriction. In this study, we addressed a critical question of whether IFITMs work by a proximity mechanism, which requires their presence at the sites of virus entry and whether the lack of local IFITMs is a major determinant of virus resistance.

Through constructing a functional fluorescently tagged IFITM3 protein, we were able to visualize its dynamic distribution in living cells, in the context of single virus entry and fusion. Imaging experiments demonstrate that IAV restriction involves virus co-trafficking with IFITM3-containing endosomes that can culminate in lipid mixing (hemifusion) but does not progress to complete fusion (viral content release). This important finding, along with our previous work [17, 30, 35], strongly supports a proximity model for virus restriction, as opposed to alternative models that involve, for example, dysregulation of cholesterol transport from late endosomes [18, 39]. Also importantly, we documented the “avoidance” mechanism of LASVpp escape from IFITM3 restriction through virus trafficking and fusion with endosomes lacking this restriction factor. Consistently, LASV GPc-mediated cell-cell fusion is sensitive to IFITM proteins expressed on the surface of target cells. These results highlight the importance of regulation of IFITM trafficking for antiviral activity and offer important clues regarding the determinants of virus resistance to restriction. Of note, the presence of IFITM3 at the sites of IAV fusion does not rule out the possibility that the antiviral effect is due to recruitment of downstream effector proteins, such as ZMPSTE24 [60]. IFITMs have the propensity to hetero-oligomerize [21] and interact with a number of other proteins [61], so it is possible that IFITM-driven protein complexes alter the membrane properties and disfavor viral fusion (see below).

Single particle tracking revealed that IAV fusion was inhibited in compartments that accumulated substantial amounts of fluorescent IFITM3. Due to the relatively high and variable background fluorescence in cells expressing fluorescent IFITM3, it is difficult to quantitatively assess whether there is a threshold density of this protein below which viruses are not restricted. In other words, it is unclear whether inhibition of IAV fusion by IFITM3 occurs through an all-or-none mechanism or there is an inhibition “gradient” whereby the probability of fusion is inversely proportional to the IFITM3 signal. Future studies using improved fluorescence labeling techniques and controlled IFITM3 expression levels will help distinguish between these modes of action. The existence of distinct domains within the highly dynamic endosomal membranes (e.g., [62–66]) adds an additional layer of complexity when interpreting the IFITM3 restriction results. It is possible that the extremely rare single LASVpp fusion events that appear to colocalize with IFITM3 occur with IFITM3-free domains within the limiting membrane of an endosome.

We have previously documented unimpeded IAV lipid mixing activity in IFITM3-expressing cells [30]. Analyses of lipid dye dequenching, irrespective of colocalization with IFITM3, did not reveal significant differences in the rate or extent of lipid mixing. This finding is in disagreement with the reduced IAV lipid dequenching in IFITM3-expressing cells reported in [39]. The reason for discrepant results is likely related to the use of a bulk lipid dequenching assay in [39], as compared to the real-time single IAV tracking in our experiments. Importantly, in the present study, we were able to show the markedly slower rate of lipid redistribution to IFITM3-containing endosomes by focusing on events occurring in these compartments compared to lipid mixing in control cells. It should be noted that, in spite of exogenous incorporation of DiI into the viral membrane in the commonly used labeling protocol (e.g., [30, 67]), the dye readily redistributes to both membrane leaflets, as we have demonstrated previously [68]. Thus, a slower lipid mixing between IAV and IFITM3-positive endosomes is consistent with a more restricted dye diffusion through the merged contacting leaflets of hemifused membranes, as compared to diffusion through both leaflets of a fusion pore.

The exact mechanism by which IFITM3 inhibits the transition from hemifusion to fusion is not clear. A large body of work demonstrates a critical role of lipid composition, and specifically of mechanical properties of lipid membranes, in protein-mediated membrane fusion (reviewed in [69]). Bending energies of highly curved lipid intermediates that form and resolve during merger of lipid bilayers are key determinants of the fusion process (reviewed in [69–71]). In addition, hemifusion and the formation of a fusion pore within a hemifusion diaphragm are associated with changes in areas of contacting and distal monolayers, respectively. Thus, viral fusion pore opening could be blocked by: (1) increased membrane bending modulus; (2) increased negative curvature of the cytoplasmic leaflet that disfavors the formation of a net positive curvature fusion pore [69, 72]; (3) expansion of the hemifusion diaphragm to a size beyond that permissible for fusion pore formation [73]; or (4) reduced “fluidity” (lateral diffusion) of the cytoplasmic leaflet, which can be caused by IFITM homo/hetero-oligomerization [21]. The latter effect is expected to disfavor the fusion pore opening due to inability to quickly remove excess lipid from the hemifusion site. IFITMs have been reported to alter membrane fluidity [21, 35], and to increase the lipid order and confer positive spontaneous curvature [22, 33]. It is thus possible that individual effects of IFITMs on lipid membranes or their combination are responsible for the fusion block. Importantly, a recent study demonstrated that mutations in distinct regions of IFITM3 regulate its inhibitory vs enhancing activity against infection by different coronaviruses [74]. The ability to switch between inhibition and promotion of coronavirus fusion by introducing point mutations in IFITM3 further supports the proximity-based mechanism of virus restriction.

We have previously proposed an alternative mechanism of IFITM3-mediated virus restriction referred to as a “fusion decoy” model [30]. According to this model, viruses are redirected into multivesicular endosomes where unrestricted fusion with intraluminal vesicles, as opposed to fusion with the limiting membrane of an endosome, does not allow viral capsid release into the cytoplasm. The single virus content (mCherry) release assay would not detect IAVpp fusion with intraluminal vesicles, as the content marker will remain contained within the same endosome. The similar extent of lipid dye dequenching upon single IAV fusion with control and IFITM3-positive endosomes (Fig 2) appears compatible with virus hemifusion to the limiting membrane, but the slower dequenching rate in IFITM3 compartments could be due to multiple rounds of hemifusion with intraluminal vesicles. Therefore, additional experiments are needed to test the validity of a “fusion decoy” model.

Recent studies have documented the ability of IFITMs to interfere with viral fusion when incorporated into the viral membrane [34, 36–38]. In fact, IFITMs appear to more potently inhibit HIV-1 infection when incorporated into virions, as compared to their expression in target cells [38]. It is tempting to assume that the same mechanism of the IFITMs’ antiviral activity is functional in both cellular and viral membranes, but this notion has not been explicitly tested. The ability of IFITM3 to inhibit IAV fusion irrespective of whether it is expressed in the target or viral membrane (Fig 8B) supports the universal mechanism of IFITM3-mediated restriction that involves altering the properties of lipid membranes, as opposed to interacting with viral or cellular proteins. Our finding that AmphoB rescues the fusion-competence of IAVpp containing IFITM3, similar to its antagonistic effect on the cell-expressed IFITM3 [35], is also consistent with the common mechanism of virus restriction. Moreover, the static nature of the viral membrane, which is in stark contrast to the highly dynamic cell membranes, supports a direct effect of AmphoB on the viral membrane, perhaps through alterations of membrane fluidity [22, 35]. Thus, virions containing IFITMs in their membranes could provide a tractable model for mechanistic studies of these proteins.

Our study focused on IFITM3 protein, which shares a relatively high sequence homology and subcellular distribution with IFITM2. Although we have not addressed the mechanism of action of the plasma membrane-resident IFITM1, the published literature and our findings support the notion that this protein also acts by a proximity-based mechanism. We thus speculate that all members of the IFITM family accumulate at the sites of fusion of sensitive viruses and block the formation of a fusion pore. The current study provided strong evidence that LASV escapes IFITM3 restriction by entering through alternative endocytic pathways, but has not addressed whether other IFITM-resistant viruses, such as Junin virus or MLV, employ the same strategy to infect IFITM-expressing cells. Future studies addressing this question will help generalize the escape mechanism discovered in this work and may suggest strategies to increase the potency of IFITMs by modulating their intracellular trafficking.

## Materials and methods

### Cell lines, plasmids and reagents

We obtained HEK 293T/17, Cos7 and human lung epithelial A549 cells from ATCC (Manassas, VA). TZM-bl cells were obtained from NIH AIDS Research and Reference Reagent Program. 293T cells stably expressing IFITM1, IFITM2 or IFITM3 were a gift from Dr. Shan-Lu Liu, Ohio State University [22]). Cells were maintained in Dulbecco’s Modified Eagle Medium (DMEM, Cellgro, Mediatech, Masassas, VA) containing 10% heat-inactivated Fetal Bovine Serum (Hyclone Laboratories, Logan, UT or Atlanta Biologicals, Flowery Branch, GA) and 1% penicillin/streptomycin from Gemini Bio-products (West Sacramento, CA). For HEK 293T/17 cells the growth medium was supplemented with 0.5 mg/ml G418 (Genesee Scientific, San Diego, CA). DMEM without phenol red was purchased from Life Technologies (Grand Island, NY). Live Cell Imaging Buffer (LCIB) and Fluorobrite^TM^ DMEM were purchased from Life Technologies (Grand Island, NY). Stable cell lines expressing fluorescently-tagged IFITM3 used for imaging analysis were obtained by transducing with VSV-G pseudotyped viruses encoding wild-type or the 2M mutant (F75/78A) IFITM3 [21] or with the Vector pQCXIN (Clontech) and selecting with 1 mg/ml G418. Following selection, cells were maintained in 0.5 mg/ml G418. Stable cell lines expressing unlabeled and fluorescently-tagged IFITM3 used for bulk fusion assays were obtained by transducing with VSV-G pseudotyped viruses encoding wild-type IFITM3 with the Vector pQXCIP (Clontech) and selecting with 1.5 μg/ml puromycin.

The pR8ΔEnv, pR9ΔEnv, BlaM-Vpr, pcRev, pMDG-VSV-G, MLV-Gag-Pol, HIV-1 Gag-mCherry (encoding an uncleavable mCherry fluorescent tag used in fixed cell co-localization experiments), IFITM3/pQCXIP, F75/78A IFITM3/pQCXIN expression vectors were described previously [15, 21, 43, 75]. The mCherry-2xCL-YFP-Vpr (mCherry fused to YFP-Vpr through a cleavable linker containing two HIV protease cleavage sites– 2xCL), as described previously [50], was used for single particle tracking of fusion events in live cells. The pCAGGS vectors encoding influenza H1N1 WSN HA and NA were provided by Drs. Donna Tscherne and Peter Palese (Icahn School of Medicine, Mount Sinai) [76]. The LASV GPc plasmid was a gift from Dr. F.-L. Cossett (Université de Lyon, France) [77].

To internally label human IFITM3 (accession NM_021034) with EGFP, sites likely permissive to insertions were identified based on sequence alignments of human and mouse IFITM family proteins. An EGFP cassette flanked by two linkers was created by PCR (forward primer TCAAGGAGGAGCACGAGGTGGCTGTGCTGGGGGCGCCCCACAACCCTGCTCCCGGCGGAGGAAGCGGCGGAGTGAGCAAGGGCGAGGAGC; reverse primer GACGACATGGTCGGGCACGGAGGTCTCGCTGCGGATGTGGATCACGGTGGATCCGCCTCCGCTTCCGCCCTTGTACAGCTCGTCCATGCC) and inserted using Gibson assembly into KasI/BsaBI-cut IFITM3 cDNA. The resulting protein has amino acids 41 (P) and 42 (T) of wild-type IFITM3 removed. The cloning of IFITM3-imNeonGreen (IFITM3-imNG), and IFITM3-imTFP1 (IFITM3-imTFP1) into pQCXIN (Clontech) and pQXCIP (Clontech) retroviral expression vectors were done in two steps. In the first step, the EGFP in IFITM3-iEGFP/pLVX Tet on construct was replaced either with mNeonGreen or mTFP1 by overlapping PCR. The IFITM3-iEGFP/pLVX Tet on construct contain an *EcoRI* restriction site in the 5’ of the IFITM3-iEGFP cDNA and a *BsrgGI* in the 3’ of GFP cDNA that facilitated the replacement of GFP. The 5’ of IFITM3 cDNA was amplified by PCR using forward primer P1 (containing *EcoRI* restriction site) TACCACTTCCTACCCTCGTAAAGAATTCGCCACCATGAATCACACTGTCCAAACCTTC, and reverse primer P2: TGTGGTCTCCTCGCCCTTGCTCACTCCGCCGCTTCCTCCGCCGGGAGC. The mTFP1 fragment was amplified using forward primer P3: GCTCCCGGCGGAGGAAGCGGCGGAGTGAGCAAGGGCGAGGAGACCACA (complementary to P2), and the reverse primer P3 (containing *BsrgGI* restriction site) GATCCGCCTCCGCTTCCGCCCTTGTACAGCTCGTCCATGCCGTCGGTGGAATT. The fragments were purified, mixed and the overlapping PCR was perfomed using the forward primer P1 and the reverse primer P3. The final PCR fragment and IFITM3-iEGFP/pLVX Tet on were digested with *EcoRI* and *BsrgGI* restriction enzymes, purified and ligated. In the second step, the IFITM3-imTFP was amplified by PCR with forward primer P4 (containing *AgeI* restriction site) GCAGGAATTGATCCGCGGCCGCACCGGTAGGCCACCATGAATCACACTGTCCAAACCTTC, and reverse primer P5 (containing *EcoRI* restriction site) AGGGGTGGGGCGGGGGGGGGCGGAATTCTTAGTGATGGTGATGGTGATGGCCTTG, digested with *AgeI* and *EcoRI* restriction enzymes, purified and ligated into pQCXIN or pQXCIP vectors. For IFITM3-imNeonGreen construct the overlapping PCR was done using IFITM3-imTFP/pQCXIN construct as template and the *AgeI and BamHI* restriction sites. The 5’ of IFITM3 cDNA was amplified by PCR using forward primer P5 (containing *AgeI* restriction site) GCAGGAATTGATCCGCGGCCGCACCGGTAGGCCACCATGAATCACACTGTCCAAACCTT, and reverse primer P6: ATCCTCCTCGCCCTTGCTCACCATTCCGCCGCTTCCTCCGCCGGGAGC. The mNeonGreen cDNA was amplified with forward primer P7: GCTCCCGGCGGAGGAAGCGGCGGAATGGTGAGCAAGGGCGAGGAGGAT (complementary to P6), and reverse primer (containing *BamHI* restriction site) CGCTGCGGATGTGGATCACGGTGGATCCGCCTCCGCTTCCGCCCTTGTACAGCTCGTCCATGCCCA. Purified PCR fragments were mixed and the overlapping PCR was done using the forward primer containing *AgeI* restriction site and the reverse primer containing *BamHI* restriction site. The fragment and IFITM3-imTFP1 plasmid were digested with *AgeI* and *BamHI*, purified and ligated. The F75/F78A IFITM3-imTFP1 (2M-IFITM3-imTFP1) mutants was obtained by Quick-change site-directed mutagenesis (Stratagen, La Jolla, CA) using IFITM3-imTFP1/pQCXIN as template.

Alexa Fluor 647-NHS ester (AF647) and the lipophilic dye SP-DiIC_18_ (1,1'-Dioctadecyl-6,6'-Di(4-Sulfophenyl)-3,3,3',3'-Tetramethylindocarbocyanine) were purchased from Invitrogen/Life Technologies (Grand Island, NY). The LASV fusion inhibitor ST-193 was purchased from Aurum Pharmatech (Franklin Park, NJ). Amphotericin B (AmphoB) was obtained from Quality Biological (Gaithersburg, MD). Primary antibodies used were rabbit directed at the N-terminus of IFITM3 (Abgent, San Diego, CA), mouse anti-tubulin from Sigma (St. Louis, MO), HIV-1 IG serum (NIH AIDS Research and Reference Reagent Program), and rabbit anti-WSN Influenza R2376 (a generous gift from Dr. David Steinhauer, Emory University). Secondary antibodies used were rabbit anti-mouse IgG(H+L)-HRP (EMD Millipore), mouse anti-rabbit IgG(H+L)-HRP (EMD Millipore), and goat anti-human IgG-HRP (H+4) (Thermo Scientific).

### Pseudovirus production and labeling

Pseudoviruses were produced by transfecting HEK 293T/17 cells with JetPRIME transfection reagent (Polyplus-transfection, Illkirch-Graffenstaden, France). For all pseudovirus productions the transfection reagent/DNA containing medium was replaced with fresh phenol red-free medium after ~14 hrs. Viruses were harvested ~48 hrs post-transfection, and cellular debris were removed by centrifuging at 230x*g* for 10 min. The collected viruses were passed through a 0.45 μm polyethersulfone filter (PES, VWR) to further clear cellular debris and virus aggregates, aliquoted and stored at -80°C. The infectious titers (~10^6^ IU/ml) were determined using serial dilutions of the inoculum in TZM-bl cells using a β-galactosidase assay. To produce the pseudoviruses for co-localization analysis, HEK293T/17 cells were grown to ~60–70% confluency in a 6-well culture dish and transfected with 0.8 μg pR8ΔEnv, 0.4 μg pcRev, 0.5 μg HIV-1 Gag-mCherry (Not cleaved by protease) and 0.8 μg GPc-Lassa or 0.4 μg each of WSN HA- and NA-expressing plasmids, respectively. For single viral fusion experiments in live cells, dual-labeled LASVpp was made by transfecting the HEK293T/17 cells grown to ~60–70% confluency in a 6-well culture dish using 0.8 μg pR9ΔEnv, 0.2 μg pcRev, 0.2 μg mCherry-2pxCLYFP-Vpr and 1 μg GPc-Lassa plasmids. Similarly, dual-labeled IAVpp were produced using 4 μg pR9ΔEnv, 1 μg pcRev, 1 μg mCherry-2xCL-YFP-Vpr and 2.5 μg each of WSN HA- and NA-expressing plasmids for transfection of ~60% confluent cells in a 100 mm dish. The purified viruses were diluted 10-fold in PBS without calcium or magnesium (PBS-/-, Cellgro, Mediatech), bound to poly-L-lysine coated 8-well chamber cover slips (LabTek, MA), and imaged to estimate the co-labeling efficiency which was over 90% for all pseudoviruses used for this study.

To generate VSV-G pseudotyped viruses encoding fluorescently-tagged IFITM3, HEK293T/17 cells grown in 6-well plate were transfected with 0.3 μg of VSV-G plasmid, 0.6 μg MLV-Gag-Pol plasmid, and 1.1 μg of either an empty pQXCIN or pQXCIP vector, or containing IFITM3-imNG, IFITM3-imTFP1, or 2M-IFITM3-imTFP1.

For intraviral IFITM3 pseudovirus production, HEK293T/17 cells grown in 100-mm dishes were transfected with 2 μg of pCAGGS H1N1 HA/NA, 3 μg pR9ΔEnv, 1.5 μg BlaM-Vpr, 0.5 μg pcRev, and 5 μg of either empty Vector pQCXIP, IFITM3, or 2M-IFITM3 using JetPRIME reagent. The viral supernatants cleared of cellular debris as described above, were concentrated 10x, using Lenti-X Concentrator (Clontech, Mountain View, CA). Following overnight concentration with Lenti-X, virus was precipitated by centrifuging at 1439x*g* for 45 min, 4°C, resuspended in DMEM without phenol red or FBS, and stored at -80°C.

For lipid mixing (hemifusion) experiments, influenza virus surface proteins and membrane were co-labeled with AF647 and with the lipophilic dye SP-DiIC_18_, respectively. Briefly, 100 μg of the purified IAV A/PR/8/34 virus (2 mg/ml, Charles River, CT) was mixed with 50 μM AF647 in 150 mM freshly prepared sodium bicarbonate buffer, pH 9.0. The labeling reaction was allowed to proceed at room temperature with tumbling in the dark for 30 min. Next, 5.8 μL of 1.75 mM SP-DiI_18_ was added to this reaction, while gently vortexing and viruses further incubated at room temperature in the dark for 1 hr with shaking. The AF647 was quenched by adding 2 μL of 1 M Tris-buffer, pH 7.0. The labeled viruses were purified from excess dyes on a Nap-5 gel filtration column (GE Healthcare) that was equilibrated with 50 mM HEPES, pH 7.4, 145 mM NaCl at room temperature. The fractions containing labeled viruses were passed through a 0.45 μm filter to remove any large lipid and/or virus aggregates. The purified viruses were bound to poly-L-lysine coverslips and imaged to quantify their co-labeling efficiency which was at least 55%, determined as the percentage of AF647 labeled viruses that showed detectable signal (under the high self-quenching concentrations) of SP-DiI_18_. The viruses were aliquoted into tubes, flash-frozen, and stored at -80°C until use.

### Virus cell fusion assay

The β-lactamase (BlaM) assay for virus-cell fusion were performed as described previously [30, 43]. Briefly, pseudoviruses containing a β-lactamase-Vpr chimera (BlaM-Vpr) were bound to target cells by centrifugation at 4°C for 30 min at 1550x*g*. Unbound viruses were removed by washing with DMEM without phenol red supplemented with 20 mM HEPES (GE Healthcare Life Sciences). Fusion was initiated by shifting to 37°^o^C for 2 hours, after which cells were placed on ice and loaded with the CCF4-AM substrate (Life Technologies) and incubated overnight at 11°C. The cytoplasmic BlaM activity (ratio of blue to green fluorescence) was measured using a SpectraMaxi3 fluorescence plate reader (Molecular Devices, Sunnyvale, CA).

### p24 ELISA and Western blotting

The p24 content of viral stocks was determined by ELISA, as described previously [78]. Whole cell lysates were harvested in RIPA Buffer (Sigma) supplemented with protease inhibitors (Complete Protease Inhibitor Cocktail, Roche), incubated on ice for 10 min, and cleared by centrifugation at 16,000x*g* for 5 min. Total protein was measured using a bicinchoninic acid assay (BCA, Pierce) and normalized protein was loaded onto 4–15% polyacrylamide gels (Bio-Rad, Hercules, CA). Precision Plus Protein Standards (Kaleidoscope Bio-Rad) were used as molecular weight markers. Proteins were transferred onto a nitrocellulose membrane, blocked in 10% Blotting-grade Blocker (Bio-Rad) in PBS-T (phosphate buffered saline with 0.1% Tween-20) for 30 min at room temperature. Membranes were incubated in primary antibodies overnight at 4°C in 5% Blotting-grade Blocker with gentle shaking: rabbit anti-IFITM3 (1:500), mouse anti-tubulin (1:3000), human HIV-IG) (1:2000), and rabbit anti-WSN Influenza R2376 (1:100). After washing membranes with PBS-T at room temperature, Horseradish peroxidase-conjugated (HRP) goat anti-rabbit, rabbit anti-mouse, and goat anti-human secondary antibodies were added in 5% Blotting-grade Buffer for 1 h at room temperature with gentle shaking. Following PBS-T washing of membranes, ECL Prime chemiluminescence reagent (GE Healthcare) was used for protein detection.

### Cell-cell fusion

The effector Cos7 cells were transfected with the Lassa virus GPc expression vector. Briefly, cells were grown on 35 mm culture dishes to ~60% confluency and transfected with 4 μg GPc expression vector using a calcium-phosphate protocol [22]. After 48 hours following transfection, cells were loaded with 1.3 μM of the green cytoplasmic Calcein-AM dye (Invitrogen). In parallel, 293T cells or their derivatives stably expressing human IFITM1, IFITM2 or IFITM3 [22] were labeled with 30 μM of the blue cytoplasmic dye CMAC (Invitrogen). Effector and target cells were washed, detached from the culture dishes using a non-enzymatic solution, resuspended in PBS++, mixed at a 1:1 ratio and co-plated onto 8-well chamber slides. After incubating for 30 min at room temperature, cells were exposed to a pH 5.0 buffer at 37°C for 20 min, and the resulting cell-cell fusion was measured by visual inspection under a fluorescent microscope, as described in [22]. Ten fields of view each containing 10–12 heterologous cell pairs were examined in each well.

### 3D co-localization of IAV and LASV with IFITM3-containing compartments

A549 cells expressing IFITM3-imNG were cultured on collagen-coated 8-chamber coverslips (Lab-Tek, NY #1.5 glass) in Fluorobrite DMEM to ~60–80% confluency. The cells were chilled by placing on ice for 10 min, followed by aspirating the media and washing with cold phosphate buffered saline with calcium and magnesium (PBS+/+). Cells were inoculated with a 5-fold dilution of the mCherry-labeled IAV or LASV pseudoviruses in 100 μL of cold LCIB supplemented with 2% FBS, and viruses were allowed to bind to cells by incubation at 4°C for 90 min. Unbound viruses were removed by washing with cold PBS+/+, and virus entry was initiated by adding 200 μL pre-warmed (37°C) LCIB. The slides were incubated at 37°C for varied times followed by fixation with 4% paraformaldehyde (PFA) in PBS-/- for 10 min at 37°C. For the zero time-point, cells were fixed with PFA immediately following the initial virus binding step at 4°C. After fixation, PFA was washed away with PBS-/- a few times and cells were imaged.

For co-localization analysis, the fixed cells were imaged on DeltaVision Elite (GE Healthcare) widefield microscope, using an Olympus PlanApoN 60x/1.42 NA oil immersion objective. Multiple Z-stacks with a spacing of 0.1 μm covering the entire thickness of the cells were acquired using a standard GFP/Cherry filter set. Deconvolution of the Z-stacks was done post acquisition to improve the signal-to-background ratio of the IFITM3-mNeonGreen vesicles and of mCherry labeled viruses using SoftWorX (DeltaVision, GE Healthcare). The deconvolved Z-stacks were used for quantitative volume based (voxel based) co-localization analysis using a custom protocol in the image analysis program Volocity (Perkin Elmer, Waltham, MA). Briefly, after background subtraction, the IFITM3 containing vesicles were identified as objects. mCherry virus particles with a size/volume threshold of 0.512 μm^3^ (corresponding to a 2x2x2 voxel) and that were associated with IFITM3-expressing cells were identified. A virus particle was considered co-localized with IFITM3 when at least 50% of its volume overlapped within an IFITM3 expressing object. For every time point, at least 4 different fields of view containing multiple cells were imaged and the mean values of the % co-localization calculated.

### Fixed cell imaging

A549 IFITM3-imNG, IFITM3-imTFP1, or 2MIFITM3-imTFP1 cells were seeded onto 35 mm collagen-coated glass-bottom Petri dishes (MatTek, MA) one day prior to imaging and cultured in DMEM without phenol red supplemented with 10% FBS, penicillin, and streptomycin. Following a wash with room temperature PBS, cells were fixed in 3.5% paraformaldehyde in PBS for 10 min. For imaging with LysoTracker™, cells were seeded as above and incubated with 30 nM LysoTracker™ Red DND-99 (ThermoFisher) diluted in pre-warmed LCIB supplemented with 2% FBS for 30 min prior to fixation. Images were acquired on a DeltaVision microscope using an Olympus UPlanFluo 40x/1.3 NA oil immersion objective (Olympus, Japan). Multiple Z-stacks with a spacing of 0.1 μm covering the entire thickness of the cells were acquired and deconvolved.

### Single-virus imaging in live cells

A549 cells were seeded onto 35 mm collagen-coated glass-bottom Petri dishes (MatTek, MA) one day before imaging and cultured in Fluorobrite DMEM supplemented with 10% FBS, penicillin, streptomycin and L-glutamine. Before imaging, the cells were pre-chilled on ice and washed with ice-cold PBS+/+. A small amount of viral suspension (~1 μL) diluted in 60 μL of cold LCIB was added to the cells and spinoculated at 1550x*g* at 4°C for 20 min. After spinoculation, the cells were washed twice with PBS +/+ to remove any unbound viruses and a small volume (~150 μL) of ice-cold LCIB was added to the cells. Viral entry was initiated by adding 2 mL of pre-warmed (37°C) LCIB containing 2% FBS, and cells were imaged immediately on DeltaVision microscope equipped with a temperature and humidity-controlled chamber. Every 6–8 sec, at least three Z-stacks spaced by 1.5–2 μm were acquired to cover the thickness of cells using Olympus 40x UPlanFluo 40x/1.3 NA oil objective (Olympus, Japan). The three-color viral fusion experiments were done with A549 cells expressing IFITM3-imTFP1 or 2M-imTFP1 using a standard CFP/YFP/Cherry filter set (Chroma, VT), while a TRITC/FITC/Cy-5 filter set was used for the three-color lipid mixing experiments with IFITM3-mNeonGreen expressing cells. LCIB in all the experiments was supplemented with 2% FBS.

### Single-virus event annotation and tracking

The time-lapse Z-stack movies are visually inspected as maximum intensity projections, using ImageJ, and the ROI manager tool was used to annotate the single fusion or hemifusion events (observed as color change or DiI intensity increase). The sets of waiting times for hemi/fusion obtained from multiple movies are combined from experiments done on different days, sorted and plotted as cumulative probability curves that show the kinetics of the event. Acquired image series were converted to maximum intensity projections and annotated particles were tracked using either Volocity (GE Healthcare) or ICY image analysis software (icy.bioimageanalysis.org). Fluorescently labeled viral particles were identified using the spot detection algorithm and tracked in 3D to determine fluorescence intensities at every time point. With three-color imaging to track double-labeled viral particles that co-traffic with fluorescently-labeled IFITM3 compartments, single Z-planes in which the viral particle trafficked were used so that background subtraction could be performed using the ICY spot tracking algorithm. The local background was determined by dilating the identified objects corresponding to viral particles by two pixels. The difference between the integrated intensities of the particle and the dilated surrounding gave the intensity surrounding the particle, from which an average per pixel local background was calculated. This was used to obtain the background-corrected intensity of the particle at every time point, which is plotted in the time traces as shown in the figures.

### Lipid dequenching (hemifusion) assay

For the lipid mixing experiments, SP-DiI_18_ dequenching curves for the individual viruses were obtained by tracking particles, either using the AF647 channel or the DiI channel. The dequenching ratios and times were manually obtained from time traces that show at least 4-fold increase in SP-DiI_18_ intensity. The dequenching ratio was calculated as ratio of the mean SP-DiI_18_ intensity before the rise of the signal and the mean intensity after completion of dequenching. The dequenching time was measured as time taken to reach an intensity plateau from the time of initial rise in intensity. The traces showing multi-phase increase in SP-DiI_18_ signal in IFITM3 expressing cells were excluded from the calculation of dequenching ratios and times.

## Supporting information

S1 Fig Expression of fluorescently labeled IFITM3 constructs in A549 cells. Stable cell lines constitutively expressing IFITM3-imNG (**A**), IFITM3-imTFP1 (**B**), or 2M-IFITM3-imTPF1 (**C**) were fixed and counterstained with Hoechst and imaged. Scale bars 27 μm. (**D**) The fraction of cells expressing IFITM3-imNG, IFITM3-imTFP1, and 2M-IFITM3-imTFP1. The total number of analyzed cells (identified by Hoechst staining) was 411, 405, and 323, respectively.
(TIF)

S2 Fig IFITM3 expression does not affect the kinetics of IAV hemifusion but can slow down lipid mixing. **(A)** Kinetics of DiI dequenching in Vector and IFITM3 expressing A549 cells. The waiting times to onset of DiI dequenching were determined by single particle tracking and plotted as cumulative distributions. **(B)** Images showing lipid mixing between IAV co-labeled with SP-DiI_18_ (green) and AF647 (red) and an endosome in A549-IFITM3-imNG (blue) cells. Dequenching of SP-DiI_18_ occurs as a result of HA-mediated lipid mixing. Scale bar 3.1 μm. **(C)** Fluorescence traces for the IAV hemifusion event in (A) that co-traffics with an IFITM3+ compartment, with a biphasic increase in intensity of SP-DiI_18_, suggesting the possibility of transient closure of the fusion pore or transition from a hemifusion structure that is more restrictive to lipid diffusion to a fusion pore. The reference AF647 signal remains steady.
(TIF)

S3 Fig IAVpp fusion can occur in the vicinity of IFITM3-positive compartments. **(A)** Time series images showing fusion of IAVpp in an IFITM3-imTFP1 expressing A549 cell. IAVpp comes in close proximity with an IFITM3+ vesicle, but does not co-traffic with it, and fusion occurs in the vicinity of the IFITM3+ endosome. **(B)** Fluorescence traces of the particle tracked in (A) show the fusion event around 15 min. *Inset*: The close-up view of the trace on the right shows that the IAV particle does not consistently (for more than 5 consecutive frames or 30 sec) co-traffic with the IFITM3+ endosome prior to fusion. **(C)** 3-dimensional trajectories of the virus and endosome marked in panel A. (See S7 Movie).
(TIF)

S4 Fig Analysis of acidity of IFITM3-imTFP1 containing endosomes. **(A)** Images of stable A549 cell lines constitutively expressing IFITM3-imTFP1 incubated with 30 nM LysoTracker Red DND-99 (LTRed) for 30 min at 37°C prior to fixation in 3.5% paraformaldehyde. The enlarged boxed area is shown on the right. Scale bar 3 μm. **(B)** Average sum intensities for each above-background IFITM3-imTFP1 and LysoTracker™ spots from 13 randomly selected cells were analyzed. The average fractions of double-positive endosomes normalized to the total IFITM3-imTFP1 or LysoTracker (LTRed) spots are shown.
(TIF)

S5 Fig Kinetics of IAVpp fusion. **(A)** Kinetics of IAVpp fusion events in Vector, IFITM3-imTFP1, 2M-IFITM3-imTFP1, and IFITM3-imTFP1 cells treated with 1 μM AmphoB. A total of 90, 12, 36, and 23 fusion events were annotated out of 4248, 4133, 2487, and 921 total viral particles in Vector, IFITM3-imTFP1, 2M-IFITM3-imTFP1, and IFITM3-imTFP1 cells treated with AmphoB, respectively. The kinetics of IAVpp fusion in IFITM3-imTFP1 cells is slower than in 2M-IFITM3-imTFP1 and IFITM3-imTFP1 cells treated with AmphoB.
(TIF)

S6 Fig Kinetics of LASVpp fusion. **(A)** Cumulative distribution of waiting times to single LASVpp fusion in A549 Vector or IFITM3-imTFP1 (IFITM3+) cells. A total of 53 fusion events were annotated in Vector cells from 2931 particles; a total of 15 fusion events occurred in IFITM3-imTFP1 cells from 1683 particles. **(B)** Kinetics of LASVpp escape from inhibition by NH_4_Cl (40 mM) added at indicated times post-infection. Virus fusion was measured by the BlaM assay. Data points are means and STD of combined duplicate measurements from two independent experiments. The somewhat slower kinetics of fusion observed by the bulk BlaM assay (B) relative to single virus imaging-based fusion kinetics (A) is likely due to the limited time of imaging and difficulties with reliable detection of late fusion events by single particle tracking.
(TIF)

S7 Fig IFITM3-containing extracellular vesicles do not modulate IAVpp fusion. (**A**) Illustration of the experimental protocol for testing the effect of extracellular medium containing cell-derived IFITM3-containing vesicles on IAV fusion (top) and measurements of IAVpp fusion with A549 cells, using a BlaM assay (bottom). Control and IFITM3 containing IAVpp (also containing BlaM-Vpr) were prepared as in Fig 8. In control experiments, HEK293T/17 cells were transfected with an empty vector or IFITM3 expressing vector. Equal volumes of superntatants collected from the mock and IFITM3 transfected cells were mixed in equal volumes with IAVpp prior to incubation with A549 cells and fusion was measured by the BlaM assay. Data are means and SEM based on two independent experiments performed in triplicate. (**B**) Western blot analysis of IAVpp and extracellular vesicles corresponding to the protocol in (A). Left: Extracellular media containing control IAVpp, IAVpp produced by IFITM3-expressing cells and/or vesicles derived from control or IFITM3-transfected cells were collected, concentrated with LentiX, as decribed in (A) lysed, subjected to SDS-PAGE and analyzed with anti-IFITM3 antibody. Middle/right: Western blots of control and IFITM3-containing viruses using anti-p24 or anti-influenza WSN antibodies.
(TIF)

S1 Movie IAV lipid mixing in A549 Vector cells. Dequenching of SP-DiI_18_ (green) upon entry of IAV co-labeled with AF647 (red) into A549 Vector cells. Increase in SP-DiI_18_ fluorescence is indicated by a white arrow around 22 min. Scale bar 4.5 μm. Movie is slowed to 0.2x speed (22–24 min) to emphasize the onset of dequenching. (Related to Fig 2B and 2C).
(AVI)

S2 Movie Dynamics of fluorescent IFITM3-positive endosomes in A549 cells. Trafficking of IFITM3-imTFP1 (blue) constitutively expressed in A549 cells. Scale bar 11 μm.
(AVI)

S3 Movie Lipid mixing in A549 IFITM3-imNG cells. Movie showing the dequenching of SP-DiI_18_ (green) upon entry of IAV co-labeled with AF647 (red) in IFITM3-imNG cells (blue). The increase in SP-DiI_18_ fluorescence is indicated by a white arrow around 35 min. Scale bar 4.5 μm. Movie is slowed to 0.2x speed from 35–38 min. (Related to Fig 2D–2F).
(AVI)

S4 Movie IAV pseudovirus fusion in A549 Vector cells. IAVpp containing A549 Vector cells were infected with mCherry-2xCL-YFP-Vpr labeled IAVpp which fuses at 30 min, as indicated by the loss of mCherry signal (white arrow). Movie is slowed to 0.2x speed to highlight the fusion event at 30–31 min. Scale bar 2.2 μm. (Related to Fig 3B and 3C)
(AVI)

S5 Movie IAV pseudovirus co-trafficking with an IFITM3-positive endosome. A549 IFITM3-imTFP1 cells were inoculated with IAVpp containing mCherry-2xCL-YFP-Vpr. Movie shows the co-trafficking of the viral particle with an IFITM3+ endosome (starting at 7:07 min), merger with additional IFITM3+ endosomes at 10:07 and 43:37 min (white arrows), but no fusion. Movie is slowed to 0.2x speed from 7–12 min and 40–45 min. Scale bar 3.4 μm. (Related to Fig 3D–3F).
(AVI)

S6 Movie IAV pseudovirus fusion outside of an IFITM3+ endosome. IAVpp was prepared as in S5 Movie. IAVpp seemingly encounters an IFITM3+ endosome at around 23 min (white arrow) but does not co-traffic with it. Fusion occurs at 31:25 without any detectable IFITM3+ signal associated with the particle. Movie is slowed to 0.2x speed at 23- and 31-min. Scale bar 3.3 μm. (Related to Fig 3G–3I).
(AVI)

S7 Movie IAV pseudovirus fusion associated with apparently transient IFITM3-positive endosome colocalization. IAVpp was prepared as in Supplemental Movies S5 and S6. IAVpp only transiently overlaps with an IFITM3+ endosome at 15:25, as indicated by the white arrow. Fusion occurs just shortly thereafter, at 15:49 but without any detectable IFITM3+ signal associated with the virus. Movie is slowed to 0.2x speed from 14–17 min. Scale bar 2.2 μm. (Related to S3 Fig).
(AVI)

S8 Movie IAV pseudovirus fusion with an IFITM3-positive endosome in cells treated with Amphotericin B. IAVpp was labeled with mCherry-2xCL-YFP-Vpr and added to A549-IFITM3-imTFP1 cells treated with 1 μM AmphoB. Movie depicts a viral particle entering an IFITM3+ endosome at around 33 min (white arrow) and co-trafficking until fusion at 42 min (loss of mCherry signal, white arrow). Movie is slowed to 0.2x speed at 34–46 and 42-44min. Scale bar 2.1 μm. (Related to Fig 4C and 4D).
(AVI)

S9 Movie IAV pseudovirus fusion in a cell expressing inactive IFITM3 mutant. IAVpp was prepared with mCherry-2xCL-YFP-Vpr and added to cells expressing the 2M-IFITM3-imTFP1 mutant. The viral particle does not co-traffic with any local IFITM3-positive maxima, and fuses at around 19 min (white arrow). Movie is slowed to 0.2x speed from 18–21 min. Scale bar 4.1 μm. (Related to Fig 4E and 4F).
(AVI)

S10 Movie LASV pseudovirus fusion in A549 cells. LASVpp was labeled with mCherry-2xCL-YFP-Vpr and added to A549 Vector cells. The particle undergoes YFP quenching at around 32 min (white arrow) and fusion at around 44 min, as indicated by the sudden loss of mCherry and de-quenching of YFP (white arrow). Movie is slowed to 0.2x speed at 32–35 min and 43–45 min. Scale bar 4.0 μm. (Related to Fig 5B and 5C).
(AVI)

S11 Movie LASV pseudovirus fusion in A549 cells expressing IFITM3-imTFP1. LASVpp does not co-traffic with IFITM3-imTFP1-positive endosomes and subsequent viral fusion occurs at sites without any local IFITM3-imTFP1 maxima. YFP quenching occurs at around 14 min (white arrow) and fusion occurs at around 46 min (white arrow), as indicated by the sudden loss of mCherry and dequenching of YFP. Movie is slowed to 0.2x speed at 13–16 min and 45–46 min. Scale bar 3.1 μm. (Related to Fig 6A and 6B).
(AVI)

S12 Movie LASV pseudovirus content release from within an IFITM3 containing endosome. LASVpp co-trafficking with an IFITM3-imTFP1-positive endosome and fusion occurring at 14:49, as indicated by the white arrow. Movie is slowed to 0.2x speed from 14–15 min. Scale bar 1.6 μm. (Related to Fig 6E and 6F).
(AVI)
