# Supplementary figures and images for: Interferon-induced transmembrane protein 3 blocks fusion of sensitive but not resistant viruses by partitioning into virus-carrying endosomes
Source: PLoS Pathog. 2019 Jan 14;15(1):e1007532. doi: 10.1371/journal.ppat.1007532 (PMC6347298; doi:10.1371/journal.ppat.1007532)

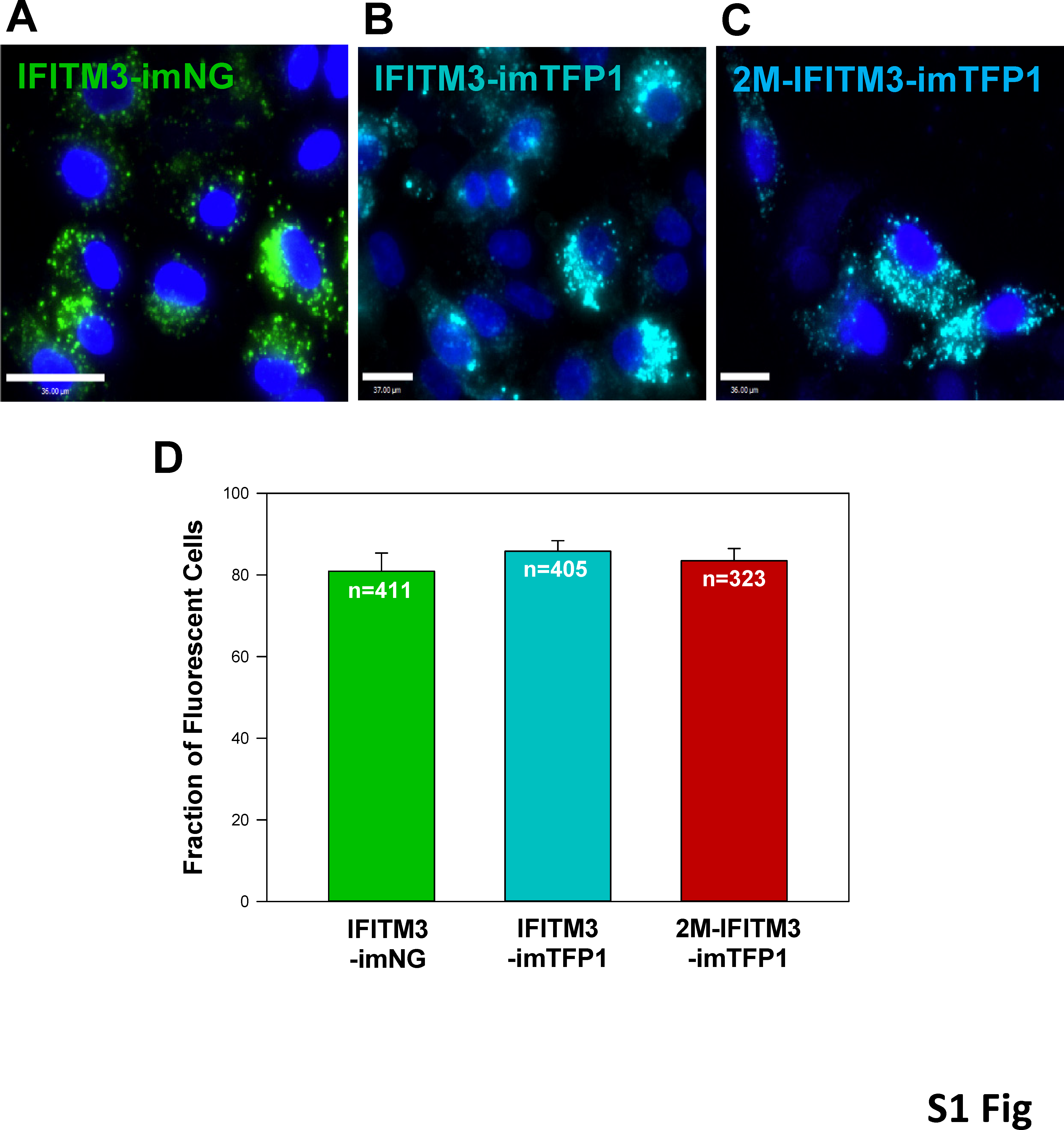

Supplement: S1 Fig — Stable cell lines constitutively expressing IFITM3-imNG (A), IFITM3-imTFP1 (B), or 2M-IFITM3-imTPF1 (C) were fixed and counterstained with Hoechst and imaged. Scale bars 27 μm. (D) The fraction of cells expressing IFITM3-imNG, IFITM3-imTFP1, and 2M-IFITM3-imTFP1. The total number of analyzed cells (identified by Hoechst staining) was 411, 405, and 323, respectively. (TIF) [file ppat.1007532.s001.tif]

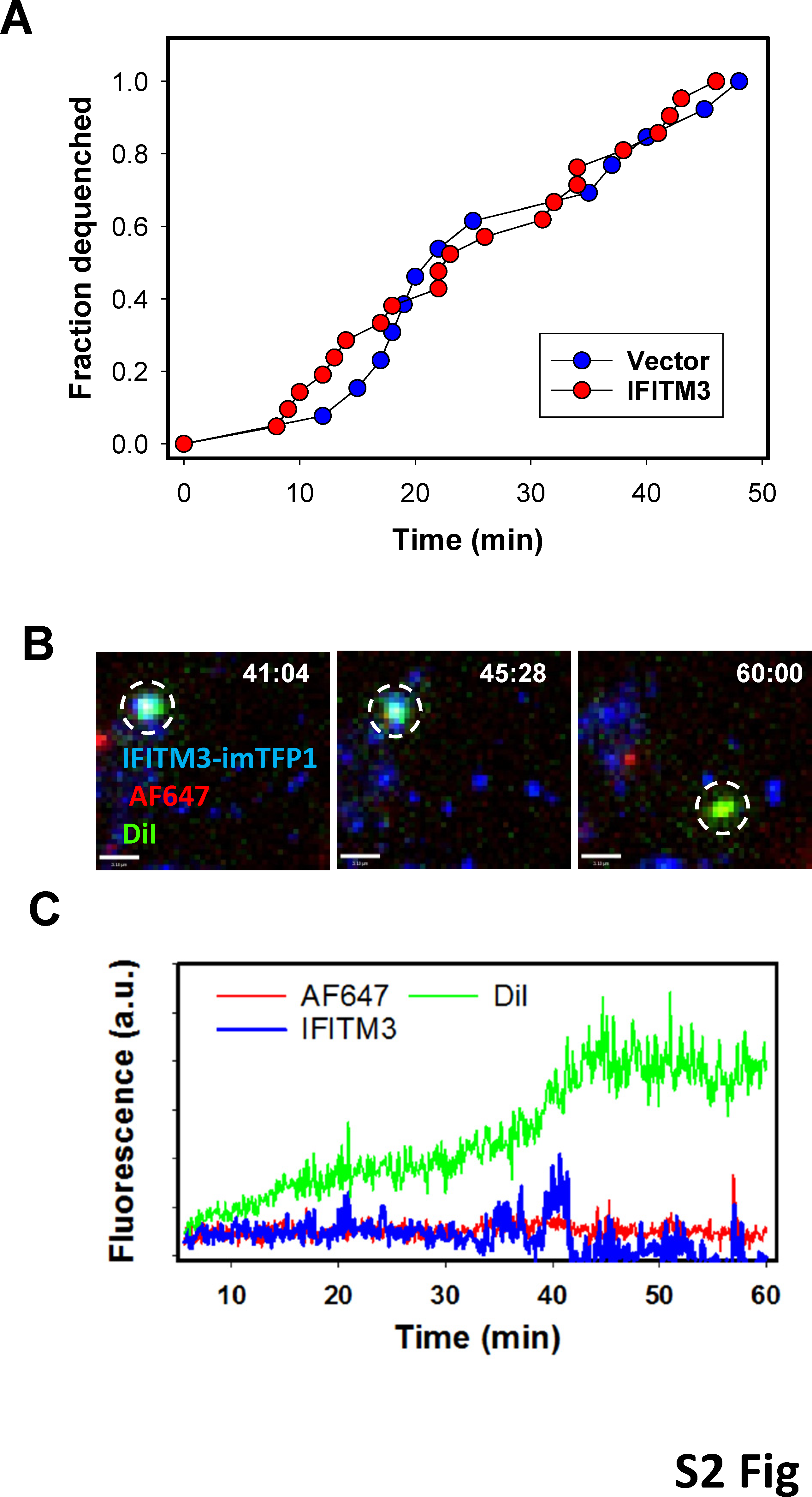

Supplement: S2 Fig — (A) Kinetics of DiI dequenching in Vector and IFITM3 expressing A549 cells. The waiting times to onset of DiI dequenching were determined by single particle tracking and plotted as cumulative distributions. (B) Images showing lipid mixing between IAV co-labeled with SP-DiI18 (green) and AF647 (red) and an endosome in A549-IFITM3-imNG (blue) cells. Dequenching of SP-DiI18 occurs as a result of HA-mediated lipid mixing. Scale bar 3.1 μm. (C) Fluorescence traces for the IAV hemifusion event in (A) that co-traffics with an IFITM3+ compartment, with a biphasic increase in intensity of SP-DiI18, suggesting the possibility of transient closure of the fusion pore or transition from a hemifusion structure that is more restrictive to lipid diffusion to a fusion pore. The reference AF647 signal remains steady. (TIF) [file ppat.1007532.s002.tif]

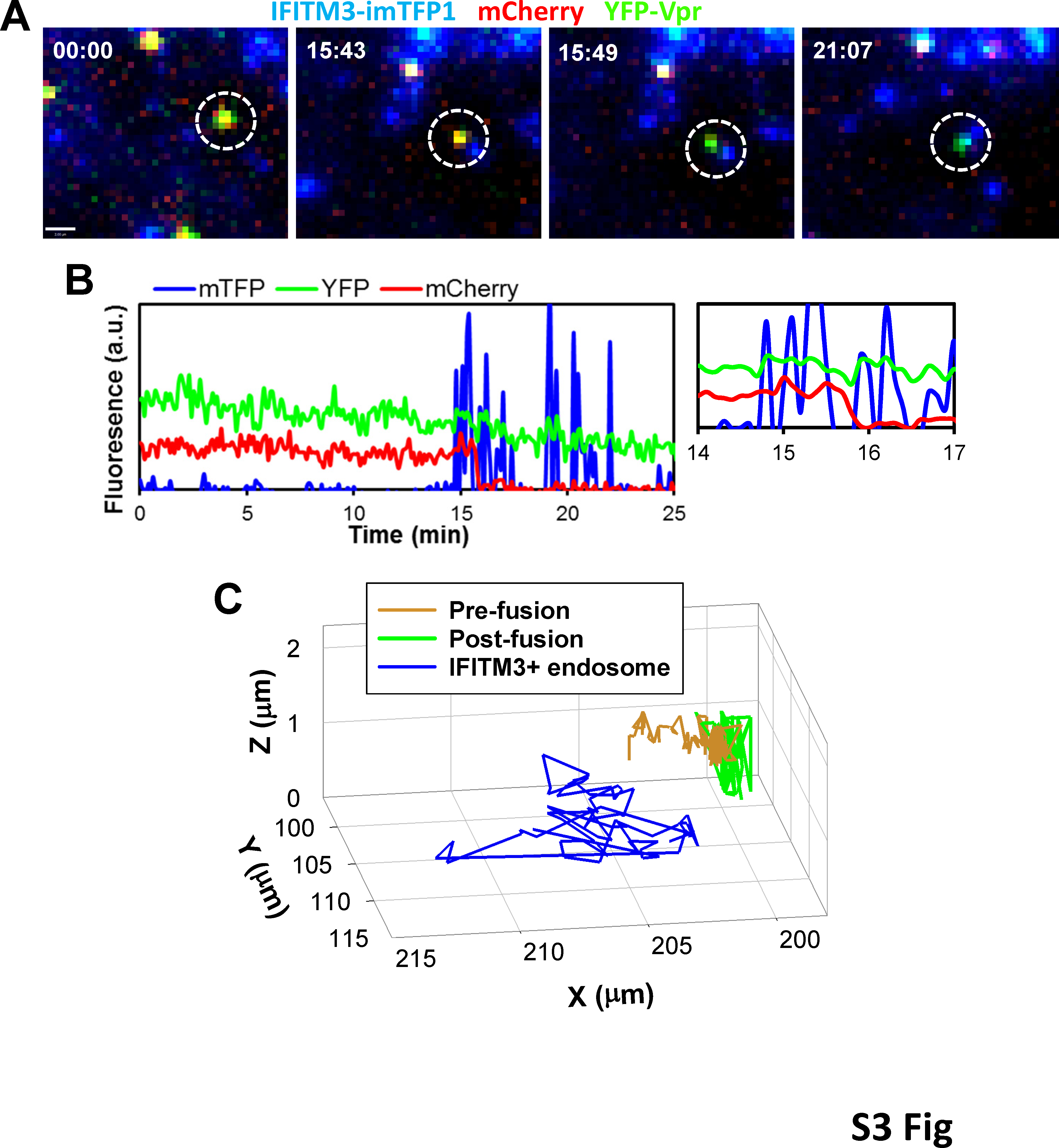

Supplement: S3 Fig — (A) Time series images showing fusion of IAVpp in an IFITM3-imTFP1 expressing A549 cell. IAVpp comes in close proximity with an IFITM3+ vesicle, but does not co-traffic with it, and fusion occurs in the vicinity of the IFITM3+ endosome. (B) Fluorescence traces of the particle tracked in (A) show the fusion event around 15 min. Inset: The close-up view of the trace on the right shows that the IAV particle does not consistently (for more than 5 consecutive frames or 30 sec) co-traffic with the IFITM3+ endosome prior to fusion. (C) 3-dimensional trajectories of the virus and endosome marked in panel A. (See S7 Movie). (TIF) [file ppat.1007532.s003.tif]

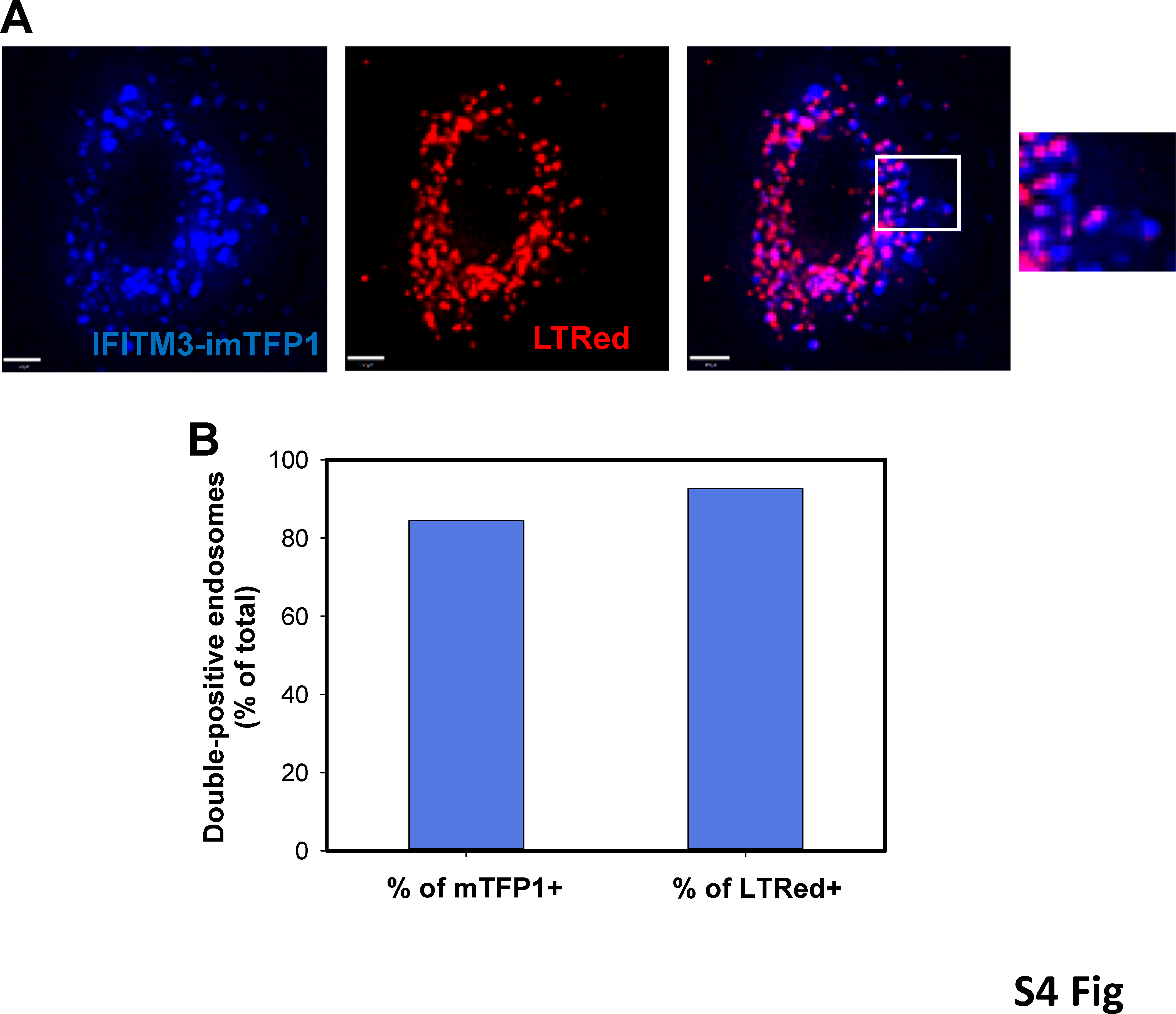

Supplement: S4 Fig — (A) Images of stable A549 cell lines constitutively expressing IFITM3-imTFP1 incubated with 30 nM LysoTracker Red DND-99 (LTRed) for 30 min at 37°C prior to fixation in 3.5% paraformaldehyde. The enlarged boxed area is shown on the right. Scale bar 3 μm. (B) Average sum intensities for each above-background IFITM3-imTFP1 and LysoTracker™ spots from 13 randomly selected cells were analyzed. The average fractions of double-positive endosomes normalized to the total IFITM3-imTFP1 or LysoTracker (LTRed) spots are shown. (TIF) [file ppat.1007532.s004.tif]

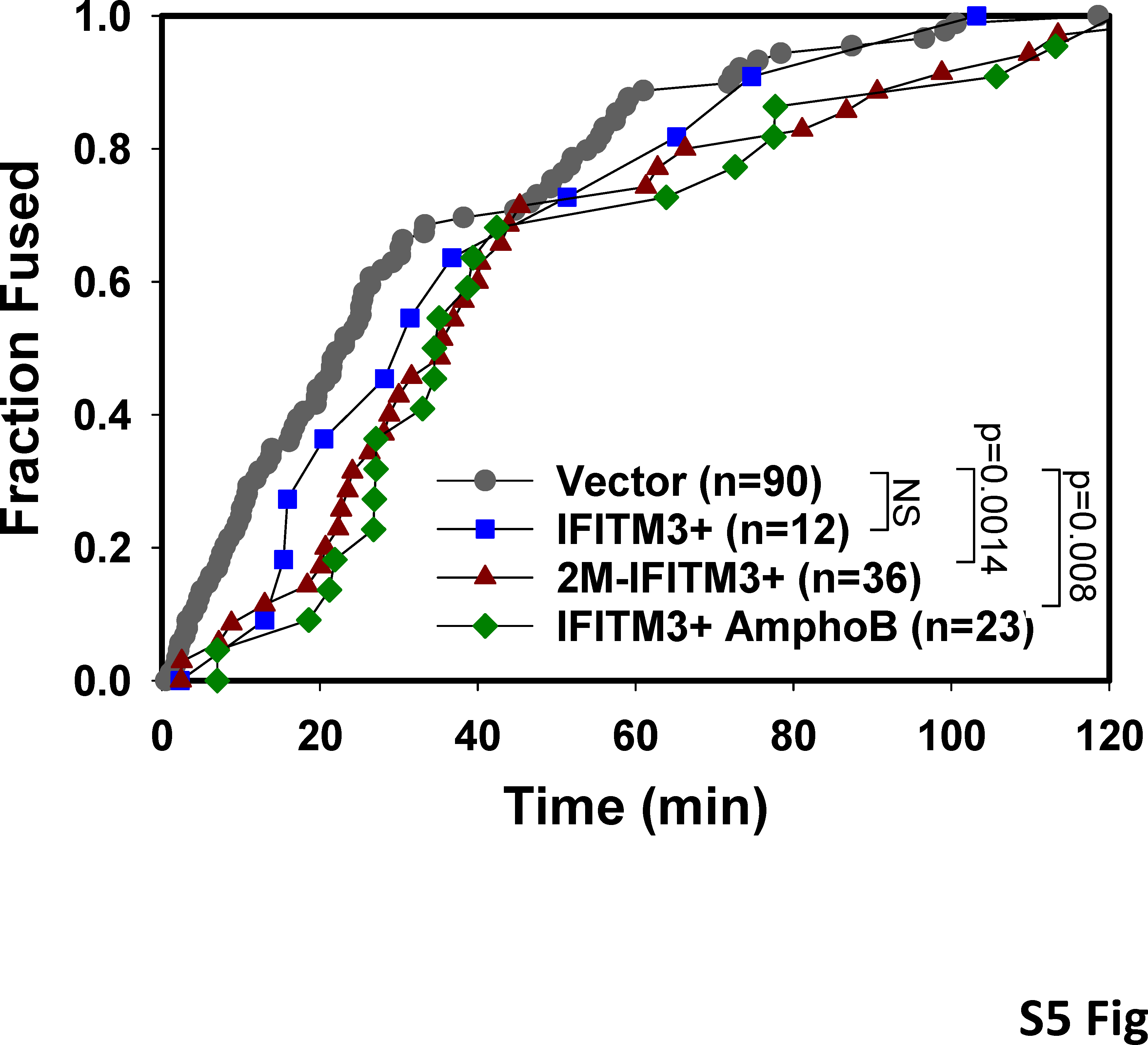

Supplement: S5 Fig — (A) Kinetics of IAVpp fusion events in Vector, IFITM3-imTFP1, 2M-IFITM3-imTFP1, and IFITM3-imTFP1 cells treated with 1 μM AmphoB. A total of 90, 12, 36, and 23 fusion events were annotated out of 4248, 4133, 2487, and 921 total viral particles in Vector, IFITM3-imTFP1, 2M-IFITM3-imTFP1, and IFITM3-imTFP1 cells treated with AmphoB, respectively. The kinetics of IAVpp fusion in IFITM3-imTFP1 cells is slower than in 2M-IFITM3-imTFP1 and IFITM3-imTFP1 cells treated with AmphoB. (TIF) [file ppat.1007532.s005.tif]

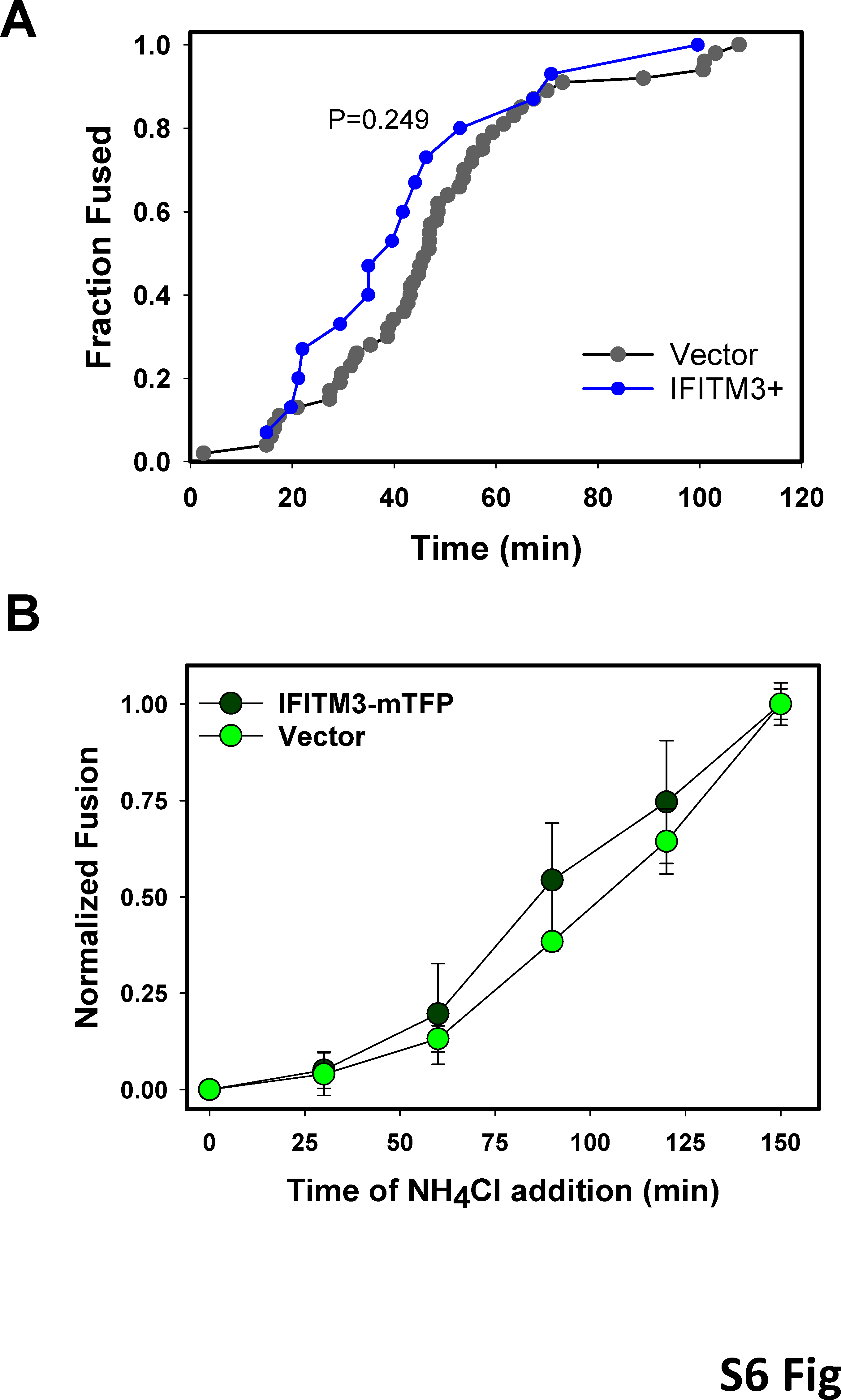

Supplement: S6 Fig — (A) Cumulative distribution of waiting times to single LASVpp fusion in A549 Vector or IFITM3-imTFP1 (IFITM3+) cells. A total of 53 fusion events were annotated in Vector cells from 2931 particles; a total of 15 fusion events occurred in IFITM3-imTFP1 cells from 1683 particles. (B) Kinetics of LASVpp escape from inhibition by NH4Cl (40 mM) added at indicated times post-infection. Virus fusion was measured by the BlaM assay. Data points are means and STD of combined duplicate measurements from two independent experiments. The somewhat slower kinetics of fusion observed by the bulk BlaM assay (B) relative to single virus imaging-based fusion kinetics (A) is likely due to the limited time of imaging and difficulties with reliable detection of late fusion events by single particle tracking. (TIF) [file ppat.1007532.s006.tif]

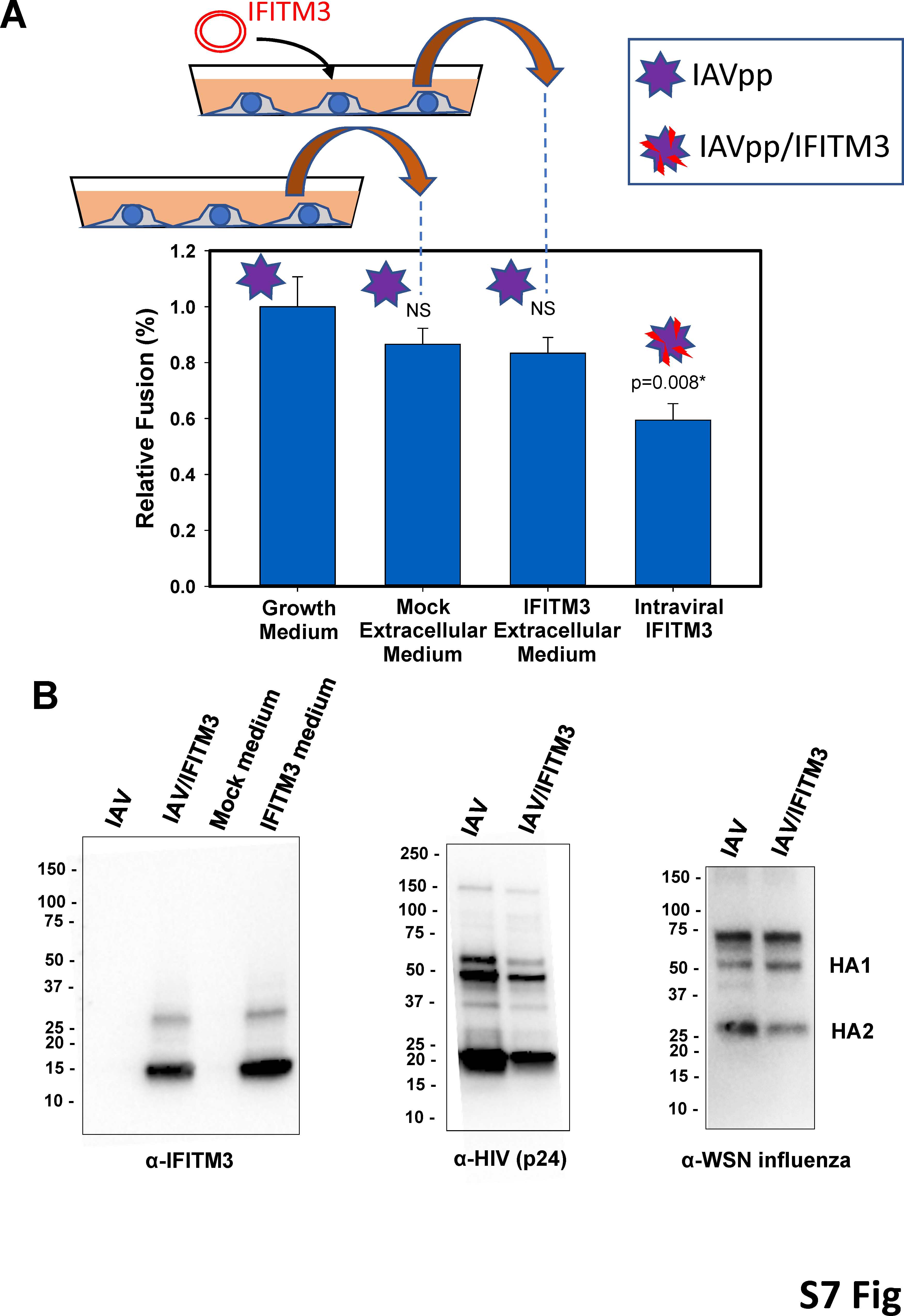

Supplement: S7 Fig — (A) Illustration of the experimental protocol for testing the effect of extracellular medium containing cell-derived IFITM3-containing vesicles on IAV fusion (top) and measurements of IAVpp fusion with A549 cells, using a BlaM assay (bottom). Control and IFITM3 containing IAVpp (also containing BlaM-Vpr) were prepared as in Fig 8. In control experiments, HEK293T/17 cells were transfected with an empty vector or IFITM3 expressing vector. Equal volumes of superntatants collected from the mock and IFITM3 transfected cells were mixed in equal volumes with IAVpp prior to incubation with A549 cells and fusion was measured by the BlaM assay. Data are means and SEM based on two independent experiments performed in triplicate. (B) Western blot analysis of IAVpp and extracellular vesicles corresponding to the protocol in (A). Left: Extracellular media containing control IAVpp, IAVpp produced by IFITM3-expressing cells and/or vesicles derived from control or IFITM3-transfected cells were collected, concentrated with LentiX, as decribed in (A) lysed, subjected to SDS-PAGE and analyzed with anti-IFITM3 antibody. Middle/right: Western blots of control and IFITM3-containing viruses using anti-p24 or anti-influenza WSN antibodies. (TIF) [file ppat.1007532.s007.tif]
